# Impact of ultrasound-assisted germination and varied drying methods on phenolic biosynthesis, antioxidant capacity, and oxidative enzyme activity in selenium-biofortified black rice

**DOI:** 10.1016/j.ultsonch.2025.107692

**Published:** 2025-11-23

**Authors:** Muhammad Tayyab Rashid, Kunlun Liu, Nazish Muzaffar, Basim M. Alohali, Mushtaque Ahmed Jatoi, Hazrat Usman, Rana Muhammad Aadil

**Affiliations:** a College of Food Science and Engineering, Henan University of Technology, Zhengzhou 450001, China; b Department of Food Science and Nutrition, University of Minnesota, 1334 Eckles Avenue, Saint Paul, MN 55108, USA; c Department of Food Science and Nutrition, College of Food and Agricultural Sciences, King Saud University, Riyadh 11451, Saudi Arabia; d Department of Botany, Shah Abdul Latif University, Khairpur 66020 Sindh, Pakistan; e National Institute of Food Science & Technology (NIFSAT), University of Agriculture, Faisalabad 38040 Punjab, Pakistan

**Keywords:** Black rice, Drying, Ultrasound, Phenolics, Volatile, Phytochemicals

## Abstract

Germinated black rice (GBR) is a nutrition-enriched product with growing consumer interest; however, drying is necessary for shelf life, which may compromise its quality. This study evaluated the effect of ultrasound (US) pretreatment combined with hot-air drying (HAD), infrared vacuum drying (IVD), and freeze-drying (FD) on the drying kinetics, phytochemicals, microstructure, and volatile composition of selenium-enriched GBR. Ultrasound significantly reduced drying time by 16.7 % in HAD and 14.8 % in IVD, with the Hii model providing the best fit for the drying kinetics (R^2^,1). Ultrasound-assisted IVD improved effective moisture diffusivity (8.11 × 10^−7^ m^2^/s) and lowered energy consumption (12.91 kWh/kg). Phytochemical retention was enhanced, with total phenolic content increased by 28–36 % and total flavonoid content by 15–24 % under HAD and IVD, while FD better preserved individual phenolic acids. Anthocyanin retention was highest in US-IVD samples (cyanidin-3-glucoside: 3075.64 µg/g), whereas carotenoids peaked in IVD without ultrasound. SEM confirmed the presence of microchannels and porosity in US-treated grains, supporting improved mass transfer. Antioxidant activity (DPPH: 1.97 mM TE/g; FRAP: 1.90 mM TE/g) was elevated in US-HAD and US-IVD, while GC–MS showed enrichment of alcohols such as 3-heptanol and suppression of aldehydes, including 2-methyl-2-pentenal. Collectively, ultrasound-assisted HAD and IVD improved drying efficiency, phytochemical stability, and aroma quality of GBR.

## Graphical abstract

**Figure:**
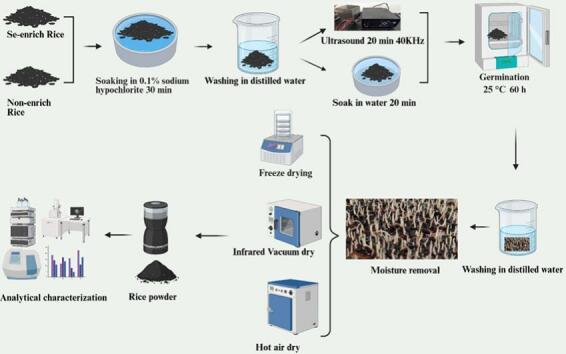

## 1 Introduction

Phytochemical-rich, pigmented rice contains bioactive compounds, including anthocyanins, proanthocyanidins, and flavonoids, which possess anti-inflammatory and antioxidant properties. Their inclusion in the diet contributes to improved health and overall well-being, particularly for individuals with gluten allergies [1]. They contain a higher concentration of active compounds that mitigate oxidative stress [2]. Based on the colour, pigmented rice can be characterised as brown, red, and black rice.

With the growing awareness of health and wellness, there is an increasing demand for foods that offer additional health benefits [3]. Improving the nutritional quality of food has become a priority in dietary research and food development. Among essential micronutrients, selenium is a critical element due to its crucial role in various physiological processes. It strengthens antioxidant defence mechanisms, regulating immune function and supports cellular health, making it an essential component of nutritional enhancement in functional food design [4]. Selenium deficiency is associated with disorders such as Keshan disease and Kashin-Beck disease [5]. In regions with low selenium levels, such as Tibet, human health has been adversely affected, highlighting the need for strategies to improve selenium intake. Germination has been recognized as a practical approach to enhance the nutritional quality of cereals, including their selenium content [6]. Germination is a traditional method used to increase grain nutrition, improving the presence of folic acid, phenolic compounds, γ-aminobutyric acid, and other bioactive substances [7]. Moreover, germination also plays a crucial role in increasing selenium levels in grain. Liu et al. (2018) [8] reported that selenium-enriched germinated brown rice contained selenium levels up to upto100 times higher after the addition of different concentrations of sodium selenite solution compared to non-enriched germinated rice. Although selenium enrichment has been widely studied in grains, such as lentils and brown rice, limited research has been reported on selenium-enriched black rice [1,8,9].

In rice processing, drying plays a significant role as a key preservation method, increasing shelf life and enhancing food availability for consumers. The main challenge is to minimize the energy usage and moisture content of the material to the optimal level without compromising flavor, color, taste, appearance, and biochemical attributes. Hot air drying (HAD) is typically used due to its cost-effectiveness. Nevertheless, the long drying time is associated with reduced quality due to nutrient depletion [10]. In the preparation of functional and ready-to-eat foods, freeze-drying or lyophilisation (FD) is generally used. It is recognized as a successful technique in the food and pharmaceutical industries for ensuring product quality and stability. Non-thermal techniques, such as ultrasound-assisted drying, are being explored as energy-efficient alternatives that can achieve comparable product quality [11,12].

To address the limitations of conventional drying practices, ultrasound-assisted drying techniques are commonly employed to reduce drying duration and preserve product attributes. It has high efficiency, non-toxicity, and eco-friendliness. Ultrasonic treatment enhances the germination rate, stimulates sprout development, and increases the levels of bioactive compounds beneficial to humans [2,13]. Ultrasound, as a mechanical wave, generates cavitation and induces structural modifications in rice grains, including the formation of internal cracks [14]. These internal cracks facilitate moisture migration, enhance water absorption, reduce cooking time and grain hardness, and improve drying efficiency [15]. The combined application of ultrasound, selenium enrichment, and advanced drying methods in black rice (Yanghei No. 3) has not been systematically studied. This work evaluates their effects on physicochemical properties, bioactive compounds, antioxidants, selenium content, and volatiles to explore the potential of black rice as a functional food ingredient.

## 2 Materials & methods

### 2.1 Source materials collection

Ordinary black rice (BR) with high germination efficiency (>85 %) and Se-enriched black rice (Se-EBR) of variety Yanghei No.3 were collected from the Henan Academy of Agricultural Science (China). The black rice was obtained by a rough rice hulled machine, followed by the separation of broken rice using a broken rice separator (FQS-13X20, Taizhou Grain Instrument Co., Ltd., China).

### 2.2 High-intensity ultrasound (HIU)

The disinfection of the samples (360 g) was performed by soaking them in 0.1 % sodium hypochlorite (500 mL) for 30 min, followed by rinsing twice with deionized water under an ultrasonic irradiator (Tianhua Co., Jining, China). Ultrasonic treatment was then applied to the disinfected rice samples at a 40 kHz simulation for 20 min and a power of 500 W/cm^3^. These parameters have been widely adopted in cereal processing due to their ability to induce cavitation and microstructural modifications while maintaining kernel integrity.

### 2.3 Germination procedure

The ultrasonically pretreated rice samples were kept in a 60-h dark period at 25 °C with 60 μM sodium selenite for germination. Samples without ultrasonic treatments were considered the control group [8]. The germination conditions followed commonly applied procedures for pigmented and selenium-enriched rice, ensuring uniform sprout development and selenium absorption. After germination, samples were thoroughly rinsed with ultrapure water to remove any residual solution before further processing.

### 2.4 Convective hot-air drying (HAD)

Se-enriched black rice ultrasound-treated samples (15 g each) were dried using a hot-air dryer at 60 °C with 1.5 m/s air velocity until equilibrium moisture content was attained. Sample weight was observed after a 20-min interval and continued until the moisture content reached 5.01 %. The selected drying temperature is commonly used for pigmented cereals to ensure effective moisture removal while limiting quality deterioration.

### 2.5 Infrared vacuum drying (IVD)

Se-enriched black rice ultrasound-treated samples were dried in a vacuum drying oven (DP33C, Yamato, Tokyo, Japan). Samples were dried on wire mesh trays at a vacuum of −0.098 mbar for 5 h at 60 °C, achieving a moisture content of less than 10 %. All experiments were performed in triplicate. These conditions provided effective infrared heating under reduced pressure, which is suitable for maintaining the structural integrity of germinated grains.

### 2.6 Freeze drying

The treated samples were arranged in petri dishes in a single layer and stored for 24 h at −25 °C (General Electric model GE 360). The freeze-drying was performed using a freeze dryer (LGJ-25E, Beijing Foring Technology Development Co.) at −40 °C & 160 Pa for 24 h. The final moisture content of the samples was determined to be 0.05 ± 0.01 g water/g, consistent with typical freeze-drying outcomes for cereal matrices.

### 2.7 Moisture content (MR)

The MR was measured by extracting samples (30 g) from the HAD drying chamber at 20-min intervals and from the IVD drying chamber at 30-min intervals for each drying treatment. The Kett Grain Moisture Meter (PM-8188-A) was used to determine the moisture content, which was repeated two to three times until a constant value was obtained using equation No. 1:

(1) $$Moisturecontent\left(w_{b}\%\right)=\frac{w-w_{d}}{w}\times 100$$

Where W and $W_{d}$ are the weights of the original and dried samples, and $W_{b}$ stands for the wet basis of the samples.

Simultaneously, the moisture removal rate ‘N’ was determined as kg moisture/min. Kg dry paddy using equation no. 2.

(2) $$N=\frac{M_{t+dt}-M_{t}}{\mathrm{dt}}$$

where $M_{t+dt}$ and $M_{t}$ is the moisture content in the grain at t+dt and time t. Moisture content was first measured on a wet basis (%) and then converted to a dry basis, expressed as (kg water/kg dry solids). The moisture ratio (MR) was calculated using Equation No. 3:

(3) $$M_{R}=\frac{{(M}_{t}-M_{e})}{{(M}_{o}-M_{e})}$$

$M_{e}$ is the equilibrium moisture content, and $M_{o}$ is the initial moisture content (%wet basis). However, the relative humidity of the drying air fluctuated continuously during the drying process; therefore, the equilibrium moisture content was calculated using Equation No. 4.

(4) $$M_{R}=\frac{M_{t}}{M_{o}}$$

Whereas the drying rate (DR) was determined using Equation (5):

(5) $$\mathrm{DR}=\frac{M_{t+\Delta t}-M_{t}}{\Delta t}$$

Where, $M_{t+\Delta t}$: moisture content at time difference, Δt: time difference between two measuring points.

### 2.8 Effective moisture diffusivity

It represents a heat transfer phenomenon in which water migrates through the food matrix or fiber structure [16]. The diffusivity, or moisture diffusion coefficient, is commonly estimated using Fick’s law of diffusion and calculated through established equations [17,18].

(6) $$\frac{\partial X}{\partial t}=D_{e}\frac{\partial^{2}}{{\partial X}^{2}}$$

The initial conditions and limits applied to equation (6) [17,19]:

(7) t=0;0<X<L

(8) $$X=X_{0,}t>0;X=0$$

(9) $$\frac{\partial X}{\partial t}=0,t>0;X=L$$

(10) $$X=X_{e}$$

For equation (6), the mathematical formula of the Fourier series is as follows, which was solved [17,18]:

(11) $$\mathrm{MR}=\frac{X-X_{e}}{X_{0}-X_{e}}=\frac{8}{\pi^{2}}\sum_{i=1}^{\infty }\frac{1}{{\left(2i-1\right)}^{2}}exp\left(-\frac{{\left(2i-1\right)}^{2}\pi^{2}D_{e}t}{{4L}^{2}}\right)$$

When the first term of the series is considered over a longer drying period, equation (6) becomes equation (7), known as the Crank model [17]:

(12) $$\mathrm{MR}=\frac{8}{\pi^{2}}exp\left(-\frac{\pi^{2}D_{e}t}{{4L}^{2}}\right)$$

To determine $D_{e}$ Equation (12) is linearized by applying a logarithmic transformation on both sides. Moisture diffusivity is then derived from the slope of the plot of Ln(MR) against drying time (t) using experimental data, with the slope subsequently substituted into the equation.

$-\frac{\pi^{2}D_{e}}{{4L}^{2}}$ (Mbegbu et al., 2021).

(13) $$\mathrm{LnMR}=Ln\frac{8}{\pi^{2}}-\frac{\pi^{2}D_{e}t}{{4L}^{2}}$$

### 2.9 Non-linear regression analysis

It was performed for drying modelling using thirteen drying modelling equations presented in the supplementary Table S1. To assess the good fit between the model and the experimental data, four statistical functions (R^2^, RMSE, RSS, and reduced χ^2^) were used. Where R^2^ indicates the proportion of variance in the experimental data explained by the model, with values closer to 1.0 reflecting stronger agreement. RMSE measures the average deviation between predicted and observed moisture ratios, while RSS represents the total squared residual error; lower values for both indicate higher predictive accuracy. Reduced χ^2^ evaluates the fit while accounting for model complexity and degrees of freedom, where smaller values denote a more reliable and unbiased representation of the drying behaviour.

### 2.10 Total energy consumption calculation (ET_HA_)

To determine ET_HA_, the total electricity applied to run the blower (E_b_) and electric energy heater (E_e_) during the drying process in the dryer was considered and calculated as the energy consumed by both heaters using Equation No. 14:

$E_{\in }=A\times v\times P_{a}\times C_{a}\times \Delta T\times t$ Eq. (14).

$E_{\in }$ = electrical energy consumed by the heater (kWh).

*A* = cross-sectional area of the drying chamber (m^2^).

*v* = air velocity inside the dryer (m/s).

$P_{a}$ = density of air (kg/m^3^).

$C_{a}$ = specific heat capacity of air (kJ/kg·°C).

ΔT = temperature difference between inlet and outlet air (°C).

*t* = total drying time (h).

Energy consumption Eq. (15):

$E_{b}=\frac{V^{3}}{16600}$ Eq. (15).

Where:

$E_{b}$ = electrical energy consumed by the blower (kWh).

*v* = air velocity inside the drying chamber (m/s).

16600 = empirical constant representing blower efficiency and power conversion

### 2.11 Determination of functional substance

#### 2.11.1 Extract preparation

For the extraction of biochemicals, the treated samples (2.5 g) were dissolved in 5 mL of 68 % (v/v) aqueous ethanol, using an ultrasonic cleaner (40 kHz, 60 min, 60 °C), followed by centrifugation for 10 min at 4000 rpm. The solvent was concentrated in a rotary evaporator at 60 °C, and the extracts were stored in glass bottles at −25 °C for further analysis.

### 2.12 Phenolic contents

#### 2.12.1 Total phenolic content (TPC)

TPC was quantified using the Folin–Ciocalteu colorimetric procedure, following the method described by Rashid et al. (2019) [20]. Briefly, 0.5 mL of the extract was mixed with 2.5 mL of 10 % Folin–Ciocalteu reagent and allowed to react for 5 min in the dark. Subsequently, 2.0 mL of 7.5 % sodium carbonate solution was added, and the mixture was incubated at room temperature for 30 min. Absorbance was recorded at 760 nm using a UV–Vis spectrophotometer, and quantification was carried out using a gallic acid calibration curve with the regression equation Y = 0.001X + 0.0001 (R^2^ = 0.9987). Results were expressed as milligrams of gallic acid equivalents per 100 g dry weight (mg GAE/100 g).

## 3 Total flavonoid content (TFC)

TFC was determined using the aluminum chloride colorimetric method. A 0.5 mL aliquot of the extract was combined with 2.0 mL of distilled water, followed by 0.15 mL of 5 % sodium nitrite [20]. After standing for 5 min, 0.15 mL of 10 % aluminum chloride solution was added. Six min later, 1.0 mL of 1 M sodium hydroxide was introduced, and the mixture was adjusted to 5 mL with distilled water. The solution was vortexed, and absorbance was measured at 510 nm. Catechin served as the standard, and quantification was performed using the calibration curve Y = 6.7186X − 1.1098 (R^2^ = 0.992). Results were expressed as milligrams of catechin equivalents per 100 g dry weight (mg CE/100 g).

## 4 Total anthocyanin content (TAC)

TAC was assessed using the pH differential method [21]. The extract was diluted separately in a pH 1.0 potassium chloride buffer and a pH 4.5 sodium acetate buffer at a 1:10 ratio. Both dilutions were allowed to equilibrate at room temperature for 15 min, protected from light. Absorbance readings were taken at 520 nm and 700 nm for each pH condition. The difference in absorbance between pH 1.0 and pH 4.5 reflects the concentration of monomeric anthocyanins. The absorbance (A) was calculated as Eq. (16):

(16) $$A=(A_{520}-A_{700}{)(pH1.0)}_{-}\left(A_{520}-A_{700}\right)\left(pH4.5\right)$$

Monomeric anthocyanins, expressed as cyanidin-3-glucoside equivalents, were calculated using the standard Eq. (17):

(17) $$\mathrm{TAC}=\frac{A\times MW\times DF\times 10^{3}}{\varepsilon \;\times 1\;}$$

where MW is the molecular weight of cyanidin-3-glucoside (449.2 g/mol), DF is the dilution factor, ε is the molar absorptivity of cyanidin-3-glucoside (26,900 L·mol^−1^·cm^−1^), and l is the path length (1 cm). Cyanidin-3-glucoside was used as the reference pigment, and the results were expressed as mg cyanidin-3-glucoside equivalents per 100 g dry weight (mg CGE/100 g).

### 4.1 Antioxidant activities (AA)

Antioxidant capacity was assessed by DPPH and FRAP assays and expressed as mM TE/g [20,22]. For the DPPH assay, 0.1 mL of the extract was mixed with 3.9 mL of a freshly prepared 0.1 mM DPPH solution in methanol. The mixture was kept in the dark at room temperature for 30 min, after which absorbance was recorded at 517 nm. The decrease in absorbance relative to the blank reflected the radical scavenging capacity of the sample. For the FRAP assay, the reagent was prepared by combining acetate buffer (pH 3.6), TPTZ solution, and FeCl_3_·6H_2_O in a 10:1:1 ratio and prewarming it to 37 °C. A 100 µL aliquot of the extract was added to 3.0 mL of the FRAP reagent and incubated at 37 °C for 30 min. Absorbance was then measured at 593 nm to determine the reducing power of the sample based on the formation of the ferrous–TPTZ complex. Trolox was used to construct the calibration curve for both assays, and antioxidant activity was calculated using the regression equation Y = 0.5533X – 0.2911 (R^2^ = 0.997). Results were expressed as mmol Trolox equivalents per gram of dry weight (mM TE/g).

### 4.2 Carotenoids analysis

Approximately 0.5 g of the powdered sample was extracted with 10 mL of acetone containing 0.1 % butylated hydroxytoluene (BHT) to prevent oxidation of the pigment. The mixture was vortexed and then centrifuged at 4000 rpm for 10 min. The supernatant was collected, and the residue was re-extracted until the solution became colorless, ensuring complete pigment recovery. All extracts were combined and adjusted to a known volume with acetone. Absorbance was measured at 450 nm and 503 nm using a UV–Vis spectrophotometer, as these wavelengths correspond to the major absorption peaks of carotenoid pigments in acetone extracts. The total carotenoid concentration was calculated using the coefficients reported by Tang, Cai, and Xu (2015) [25] and quantified in micrograms per gram (µg/g) using equation (18).

(18) $$\mathrm{Totalcarotenoidscontent}=4.642\times A_{450}-3.091\times A_{503}$$

### 4.3 HPLC analysis

Phenolic acids, flavonoids, and anthocyanins were quantified using a Waters HPLC system (Model E2695, Waters Corporation, USA) equipped with a photodiode array (PDA) detector [1]. Separation was achieved on a reverse-phase C18 analytical column (4.6 × 250 mm, 5 µm particle size). The mobile phase consisted of solvent A (0.1 % formic acid in water) and solvent B (acetonitrile). A gradient elution program was applied, starting with 5 % B, increasing to 15 % B over 10 min, then to 30 % B over the next 15 min, and reaching 50 % B by 35 min, followed by a re-equilibration step. The flow rate was maintained at 1.0 mL/min, the injection volume was 20 µL, and the column temperature was set at 30 °C. The PDA detector was set to monitor multiple wavelengths corresponding to each compound class: 280 nm for phenolic acids, 360 nm for flavonols, and 520 nm for anthocyanins. Identification of individual compounds was based on comparison of retention times and UV–Vis spectra with those of authenticated external standards. Quantification was performed using calibration curves constructed from standard solutions of known concentrations, and results were expressed as micrograms per gram of dry weight (µg/g). Each sample was analyzed in triplicate to ensure analytical precision.

### 4.4 Enzyme extraction

Enzyme extraction was performed according to the procedure described by Sarpong et al. (2019) [10]. Approximately 1.0 g of finely ground sample was homogenized in 10 mL of ice-cold 0.1 M phosphate buffer (pH 6.8) containing 1 % (w/v) polyvinylpyrrolidone (PVP) to prevent oxidation of phenolic substrates. The mixture was vortexed thoroughly and kept on ice during extraction to minimize enzyme denaturation. The homogenate was centrifuged at 10,000 rpm for 20 min at 4 °C, and the resulting supernatant was collected as the crude enzyme extract. All extractions were performed under chilled conditions to maintain enzyme stability.

#### 4.4.1 Enzyme activities

Polyphenol oxidase (PPO) activity was determined by monitoring the formation of quinones using catechol as the substrate [10,23]. For each assay, 0.1 mL of the crude enzyme extract was added to 2.9 mL of 0.1 M phosphate buffer (pH 6.8) containing 50 mM catechol. The reaction was initiated immediately after mixing, and the increase in absorbance at 420 nm was recorded for 3 min at 25 °C using a TU-1810 spectrophotometer. PPO activity was expressed as the change in absorbance per minute under the assay conditions. Peroxidase (POD) activity was evaluated using guaiacol as the electron donor. The reaction mixture consisted of 2.8 mL of phosphate buffer (pH 6.8) with 20 mM guaiacol and 10 mM hydrogen peroxide, to which 0.2 mL of enzyme extract was added. The formation of tetraguaiacol was measured by tracking the increase in absorbance at 470 nm for 3 min at 25 °C. POD activity was expressed based on the rate of absorbance increase per min in Eq. (19).

(19) $$Relativeactivity\left(\%\right)=\frac{\mathrm{Currentenzymeactivity}}{\mathrm{Initialenzymeactivity}}\times 100$$

#### 4.4.2 Browning analysis (BI)

Browning intensity was assessed using the procedure described by Sarpong et al. (2018) [10]. A portion of the crude enzyme extract obtained during the extraction step was diluted with 0.1 M phosphate buffer (pH 6.8) to an appropriate concentration. The diluted solution was transferred to a quartz cuvette, and absorbance was measured at 420 nm using a TU-1810 UV–Vis spectrophotometer. The absorbance value at this wavelength reflects the accumulation of brown pigments formed through enzymatic and non-enzymatic reactions in the sample. Browning index was expressed directly from the corrected absorbance reading after subtracting the corresponding blank prepared with the extraction buffer in Eq. (20).

(20) $$\mathrm{BI}=\frac{100(x-0.31)}{0.17}$$

#### 4.4.3 Microstructure analysis (SEM)

Microstructural changes in the samples during drying treatments were observed using an SEM (JEOL JSM-5800LV). The samples were coated in gold using a Sputter Coater (BALZERS, model SCD050).

#### 4.4.4 Statistical analysis

Analysis of variance (ANOVA) was performed employing Tukey’s Test at *P < 0.05, and ± SD was determined by Tukey's tests at p < 0.05 and extremely significant when **P < 0.01, being marked with one or two asterisks, respectively. Multivariate statistical analyses, including principal component analysis (PCA), Pearson’s correlation, and hierarchical cluster analysis (HCA), were conducted in Origin Pro 2024 to evaluate associations among phenolic compounds, antioxidant indices, and enzymatic activities. Each experiment was performed in triplicate.

## 5 Results and discussion

### 5.1 Drying kinetics curves

**Fig. 1:**
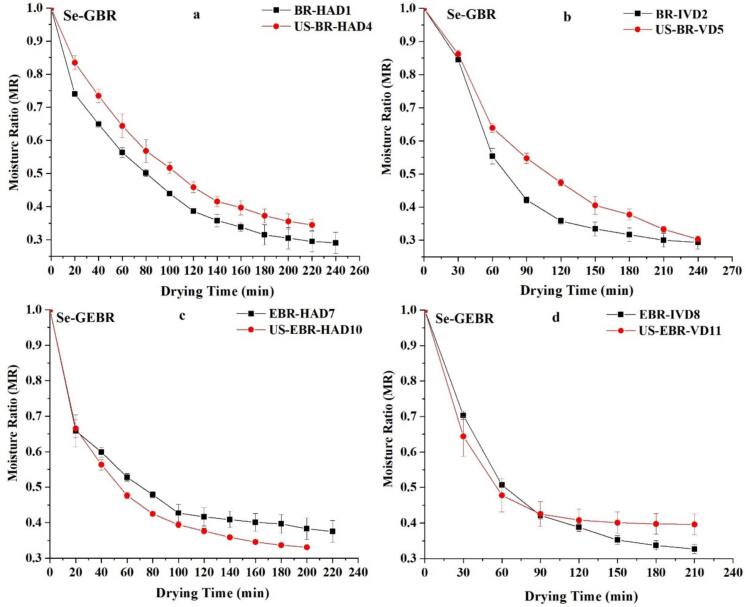
Moisture ratio of Se-GBR and Se-GEBR during hot air drying (HAD) and infrared vacuum drying (VD) with and without ultrasound pretreatment.

The drying kinetics of germinated black rice (GBR) under IVD at 60 °C and HAD with ultrasound pretreatment (40 kHz for 20 min) are presented in Fig. 1, Fig. 1. The attained drying curves for BR at 60 °C were observed to be similar to those of most food items, indicating that moisture loss is temperature-dependent [24]. The moisture content of BR was observed to decrease exponentially with increasing drying time; however, in ultrasound-pre-treated samples, the reduction in moisture occurred more rapidly. This phenomenon has been documented in the drying of various food materials [25,26]. The moisture content of the grains decreased progressively over time. The results consistently demonstrate that ultrasound pretreatment significantly reduces drying times across all conditions (p < 0.05). In HAD, the moisture ratio (MR) of unsonicated samples (BR-HAD and EBR-HAD) decreased from 1.0 to 0.3 within 240 min. In contrast, the ultrasound-treated samples (US-BR-HAD and US-EBR-HAD) achieved a lower MR of 0.25 within the same duration, reflecting an approximate 16.7 % reduction in drying time (Fig. 1, Fig. 1). The application of ultrasound induces rapid expansion and contraction of plant cells, generating bubbles in samples and their surroundings. The resulting high pressure causes changes in surface tension, which in turn affects plant cell viscosity, leading to the formation of microscopic channels and a reduction in drying time [1]. Similarly, in IVD (Fig. 1, Fig. 1), the MR of untreated samples (BR-IVD and EBR-IVD) decreased to 0.3 at 270 min. Whereas ultrasound-treated samples (US-BR-IVD and US-EBR-IVD) reached a lower MR of 0.25 in the same period, further highlighting the enhanced drying efficiency provided by ultrasound pretreatment. These observations are consistent with the drying kinetics of ultrasound-assisted drying of lentils and rice [9,27]. Such rapid drying processes are considered preferable since they not only reduce production costs and time but also maintain the quality of the products, especially in relation to temperature effects on the dried product.

### 5.2 Drying rate

**Fig. 2:**
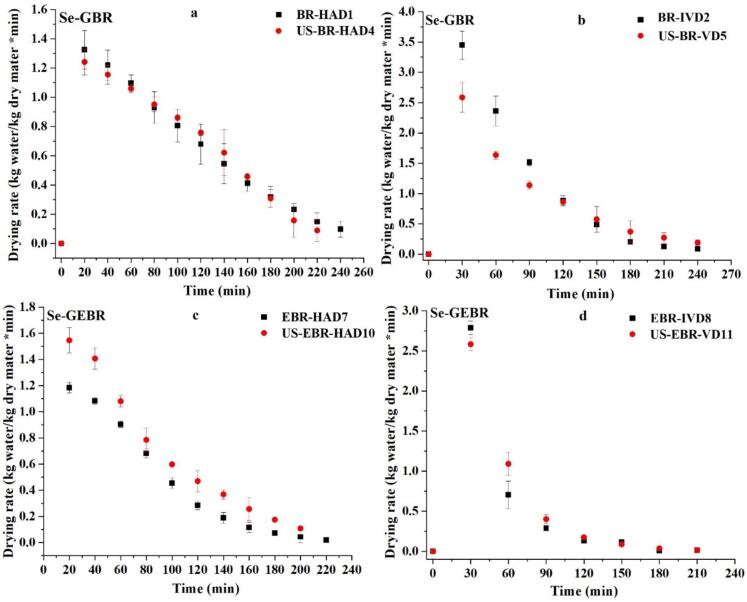
Drying rate of Se-GBR and Se-GEBR during hot air drying (HAD) and vacuum drying (VD) with and without ultrasound pretreatment.

The connection between drying rate and temperature for BR at 60 °C in relation to moisture content is presented in Fig. 2, Fig. 2. The results demonstrate a consistent decrease in drying rates over time across all samples, indicating a progressive reduction in moisture content during the drying process. Ultrasound-treated samples exhibited significantly higher drying rates (p < 0.05) compared to unsonicated samples, particularly during the initial stages of drying, highlighting the effectiveness of ultrasound pretreatment in enhancing moisture removal. In HAD (Fig. 2, Fig. 2), the initial drying rate of BR-HAD was approximately 1.4 kg water/kg dry matter/min. In contrast, the ultrasound-treated samples, US-BR-HAD, exhibited a higher drying rate of 1.6 kg water/kg dry matter/min, reflecting a 14.3 % increase. Comparable results were observed for EBR-HAD and US-EBR-HAD, with the ultrasound-treated sample demonstrating a higher drying rate during the initial stages of drying. Similar findings were noted in the case of unsonicated and sonicated black rice grains [1] and barley [5]. This improvement may be attributed to the mechanical action of ultrasound, which disrupts cellular structures, enhances water diffusivity, and lowers internal resistance to moisture migration (Santacatalina et al., 2014). As drying progressed, however, the drying rates of the ultrasound-treated and untreated samples gradually converged, suggesting that the effect of ultrasound is most evident during the initial stages of drying, when the moisture content is higher. Likewise, in IVD (Fig. 2, Fig. 2), the early drying rates were higher than those observed in HAD at 60 °C, with BR-IVD and US-BR-IVD showing initial rates of about 3.2 and 3.5 kg water/kg dry matter/min, respectively, representing a 9.4 % increase in the ultrasound-assisted sample. Comparable findings were reported by Lekcharoenkul et al. (2014) [28], who noted that the drying rate of cabbage leaves at 60 °C was substantially greater than at 45 °C. A similar enhancement was observed in EBR-IVD and US-EBR-IVD, where ultrasound treatment resulted in higher drying rates. The elevated drying rates in vacuum drying are likely due to the reduced atmospheric pressure, which lowers the boiling point of water and accelerates evaporation [29].

### 5.3 Drying kinetic modelling

**Fig. 3:**
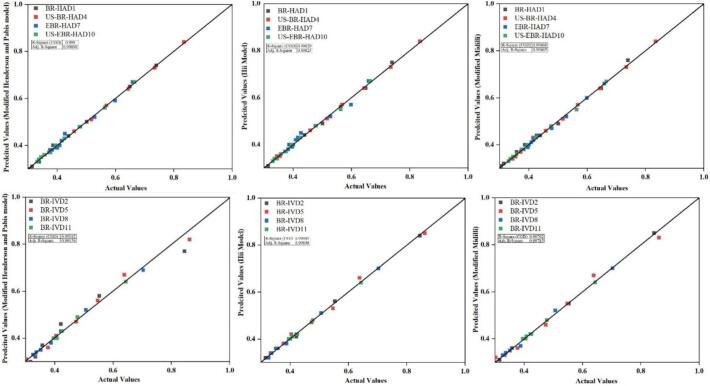
Validation of drying models by regression between actual and predicted moisture ratio values for selenium-biofortified black rice under different treatments.

To evaluate the effect of ultrasound on moisture transfer in selenium-enriched germinated black rice, experimental drying data were fitted to thirteen thin-layer models that consider external resistance, as presented in Table 1. In contrast, less significant models are presented in supplementary Table S2. [30]. All models provided acceptable agreement with experimental values, but the Hii model consistently achieved the best fit. The validation plots further confirmed the authenticity and reliability of the applied models, as shown in Fig. 3. In US-EBR-IVD11, the Hii model yielded an R^2^ value of 1.000 with an RSS of 8.28 × 10^−6^ and an RMSE of 0.0030. At the same time, similar results were obtained in other ultrasound-pretreated samples, confirming its suitability for representing modified moisture transfer during the drying process. These findings align with reports by Rashid et al. (2023) [1], which indicates that the Hii model accurately describes the drying behaviour in cereal systems. The Modified Henderson and Pabis model also showed strong fitting performance, with R^2^ values approaching 1.000 for US-RBR-HAD4 and χ^2^ as low as 2.96 × 10^−5^ for US-EBR-IVD11, supporting its applicability to ultrasound-assisted drying, as also noted by Bhargava et al. (2021). The Modified Midilli model provided satisfactory results in selected cases, such as BR-IVD2, where R^2^ reached 1.000 and RSS was 1.54 × 10^−5^. However, its performance was less consistent overall compared with the Hii and Modified Henderson and Pabis models. The high accuracy of the Hii model can be explained by its ability to capture changes in both internal and external resistance to moisture transfer. Ultrasound induces cavitation, which disrupts cell walls, forms microchannels, and enhances both diffusion and surface evaporation [31]. In contrast, germination weakens cell networks and increases enzymatic activity, further facilitating moisture removal [32]. Drying at 60 °C under infrared vacuum showed slightly better modelling accuracy than hot air, as reduced pressure lowered the boiling point of water and accelerated evaporation [33]. In particular, US-EBR-IVD11 exhibited the lowest RSS and RMSE values among all treatments, demonstrating that ultrasound pretreatment combined with vacuum drying improved the drying process. These results align with the findings of Chemat et al. (2017) [14], who reported that ultrasound-assisted drying enhances drying rate and energy efficiency while maintaining product quality. Overall, the Hii model was identified as the most reliable descriptor of moisture removal in germinated black rice, as it successfully captured the combined effects of ultrasound pretreatment, germination, and drying method on drying behaviour..

**Table 1:** Averages of selected models fitted to the ultrasound-treated selenium-enriched germinated black rice.

Sample Codes	Coefficients	R^2^	RSS	χ^2^	RMSE
	Hi Model				
RBR-HAD1 RBR-VD2 US-RBR-HAD4 US-RBR-VD5 EBR-HAD7 EBR-VD8 US-EBR-HAD10US-EBR-VD11	a. 7.3500 × 10^-005^, k1. 0.1750, n. 0.6680, b. 0.9980, k2. 0.3800 a. 0.6270, k1. 0.0000, n. 1.9520, b. 0.3760, k2. 6.1400 × 10^-006^ a. 0.9930, k1. 0.0130, n. 0.0070, b. 0.0070, k2. 0.0260 a. 0.4460, k1. 0.0010, n. 1.7000, b. 0.5570, k2. 5.6700 × 10^-005^ a. 0.9350, k1. 2.2700, n. 0.0350, b. 0.6960, k2. 2.6970 a. 0.5750, k1. 0.0090, n. 1.2750, b. 0.4250, k2. 0.0000 a. 0.9060, k1. 0.0740, n. 0.6320, b. 0.0940, k2. 0.3000 a. 0.5580, k1. 0.0160, n. 1.1980, b. 0.4120, k2. 6.6800 × 10^-005^	0.9990 0.9990 1.0000 0.9980 0.9740 1.0000 0.99901.0000	0.0000 0.0000 0.0200 0.0000 0.0000 0.0000 0.00000.0000	4.8500 × 10^-05^ 3.6000 × 10^-05^ 0.0019 0.0001 0.0002 2.2410 × 10^-05^ 3.3000 × 10^-05^ 8.2800 × 10^-06^	0.0064 0.0052 0.0387 0.0112 0.0138 0.0050 0.00570.0030
	Modified Henderson and Pabis model	R^2^	RSS	χ^2^	RMSE
RBR-HAD1 RBR-VD2 US-RBR-HAD4 US-RBR-VD5 EBR-HAD7 EBR-VD8 US-EBR-HAD10US-EBR-VD11	a. 0.1390, k. 1.0720, b. 0.4600, g. 0.0060, c. 0.8150, h. 0.0080 a. 0.9810, k. 0.0110, b. 0.0240, g. 0.0060, c. 0.0250, h. 0.0060 0. 0390, k. 0.9680, b. 0.9050, g. 0.0080, c. 0.0550, h. 0.0050 a. 0.8900, k. 0.0090, b. 0.0620, g. 0.0020, c. 0.6200, h. 0.0020 a. 0.2250, k. 1.1880, b. 0.4890, g.0.0130, c. 0.2870, h. 0.0010 a. 0.6920, k. 020, b. 0.1550, g. 0.0000, c. 0.1550, h. 0.0000 a. 0.1630, k. 1.0610, b. 0.4960, g. 0.2100, c. 0.3410, h. 0.0000 a. 0.6330, k. 0.0290, b. 0.1840, g. 0.0000, c. 0.1840, h. 0.0000	1.0000 0.9810 1.0000 0.9930 0.9980 0.9990 1.00000.9990	0.0000 0.0100 0.0200 0.0000 0.0000 0.0000 0.00000.0000	2.5300 × 10^-05^ 0.0013 0.0019 0.0003 0.0001 5.5910 × 10^-05^ 1.5920 × 10^-05^ 2.9610 × 10^-05^	0.0046 0.0324 0.0387 0.0192 0.0094 0.0079 0.00390.0079
	Modified Midilli	R^2^	RSS	χ^2^	RMSE
RBR-HAD1 RBR-VD2 US-RBR-HAD4 US-RBR-VD5 EBR-HAD7 EBR-VD8 US-EBR-HAD10US-EBR-VD11	a. 0.7920, k. 0.0360, n. 0.7630, b. 0.2050, a. 0.6590, k. 1258.9200, n. −1.8810, b. 0.9260 a. 0.7240, k. 0.0130, n. 0.9660. b. 0.2730 a. 0.7200, k. 0.0050, n. 1.1740, b. 0.2870 a. 0.3020, k.1256.6840, n. −1.7970, b. 0.6590 a. 0.0673, k. 0.0150, n. 1.0760, b. 0.3280 a. 0.6990, k. 0.7600, n. 0.7050, b. 0.3010 a. 0.6020, k. 0.0180, n. 1.1460, b. 0.3980	0.9970 1.0000 0.9990 0.9940 0.9960 0.9990 0.99601.0000	0.0000 0.0000 0.0200 0.0000 0.0000 0.0000 0.00000.0000	0.0001 1.5400 × 10^-05^ 0.0017 0.0002 5.3410 × 10^-05^ 5.8630 × 10^-05^ 2.8510 × 10^-05^ 8.2820 × 10^-06^	0.0093 0.0034 0.0365 0.0161 0.0059 0.0081 0.00530.0030

### 5.4 Moisture effective diffusivity

The effective diffusivity (Deff) of black rice during HAD and IVD was in the order of 10^−7^ m^2^/s, consistent with values reported for biologically active dried products (Table 2) [34]. Infrared vacuum drying exhibited slightly higher diffusivity than hot-air drying, as evident in BR-IVD2 (8.43 × 10^−7^ m^2^/s) compared with BR-HAD1 (7.95 × 10^−7^ m^2^/s), indicating faster moisture transfer under reduced pressure (Jimoh et al., 2023). Ultrasound pretreatment further enhanced Deff, with US-BR-IIVD5 (8.11 × 10^−7^ m^2^/s) and US-BR-HAD4 (7.78 × 10^−7^ m^2^/s) exceeding the untreated controls, consistent with cavitation-induced microchannel formation and reduced resistance to water migration [13,22,35]. The highest model fit was observed in US-BR-IVD5 (R^2^ = 0.9704), indicating a strong agreement between experimental and predicted values. In contrast, a lower correlation was observed in US-EBR-IVD11 (R^2^ = 0.6954), suggesting that structural heterogeneity was introduced by germination. These findings indicate that vacuum drying, combined with ultrasound, enhances diffusivity. At the same time, selenium enrichment modifies the grain structure in ways that sometimes reduce model accuracy, supporting earlier observations on the dual role of ultrasound in facilitating mass transfer and compensating for structural barriers [36].

**Table 2:** Effect of ultrasound on drying of selenium-enriched germinated black rice regarding moisture effective diffusivity, and specific energy consumption.

**Samples**	**Moisture diffusivity (m^2^/s)**	**R^2^**	**Energy Consumption** **(kWh/kg)**
BR-HAD1	7.95×^-07^	0.9228	14.08
BR-VD2	8.43×^-07^	0.8635	14.08
US-BR-HAD4	7.78×^-07^	0.9612	12.91
US-BR-VD5	8.11×^-07^	0.9704	14.08
EBR-HAD7	5.84×^-07^	0.7794	12.91
EBR-VD8	8.11×^-07^	0.8611	12.32
US-EBR-HAD10	7.62×^-07^	0.8234	11.73
US-EBR-VD11	6.16×^-07^	0.6954	12.32

### 5.5 Total energy consumption

The energy consumption data for ultrasound-treated and untreated GBR, dried at 60 °C using HAD and IVD, highlight the impact of ultrasound pretreatment on enhancing the efficiency of the drying method (Table 2). BR-HAD1 and BR-IVD2 exhibited energy consumption of 14.08 kWh/kg for both drying methods. However, ultrasound pretreatment notably decreased energy usage in HAD, as seen in US-BR-HAD4, where the energy consumption dropped to 12.91 kWh/kg. This reduction reflects the efficiency gains observed in a previous study [37], where ultrasound pretreatment at optimal conditions resulted in a 17.3 % decrease in total energy requirements. Similarly, in IVD, a similar trend was observed, with US-BR-IVD5 consuming 14.08 kWh/kg, indicating marginal energy savings compared to BR-IVD2. The reduction in energy consumption can be attributed to ultrasound-induced cavitation, which enhances moisture diffusivity, accelerates water removal, and shortens the overall drying duration. Using ultrasound before convective drying under suitable conditions has been reported to reduce overall energy requirements. Rashid et al. (2023) [1] found that pretreating black rice with ultrasound before drying lowered energy consumption and, in turn, reduced the production cost of germinated rice products. In this study, the average specific energy (SE) values were lower than those documented for other products dried by conventional techniques, including mushrooms dried by HAD (47.88–93.45 kWh/kg) and IIVD (41.97–124.34 kWh/kg) [38]. The relatively low SE values obtained can be explained by more efficient heat transfer and faster moisture removal, particularly under HAD and IVD, where ultrasound pretreatment provided an additional advantage by enhancing energy utilization.

### 5.6 Microstructure

**Fig. 4:**
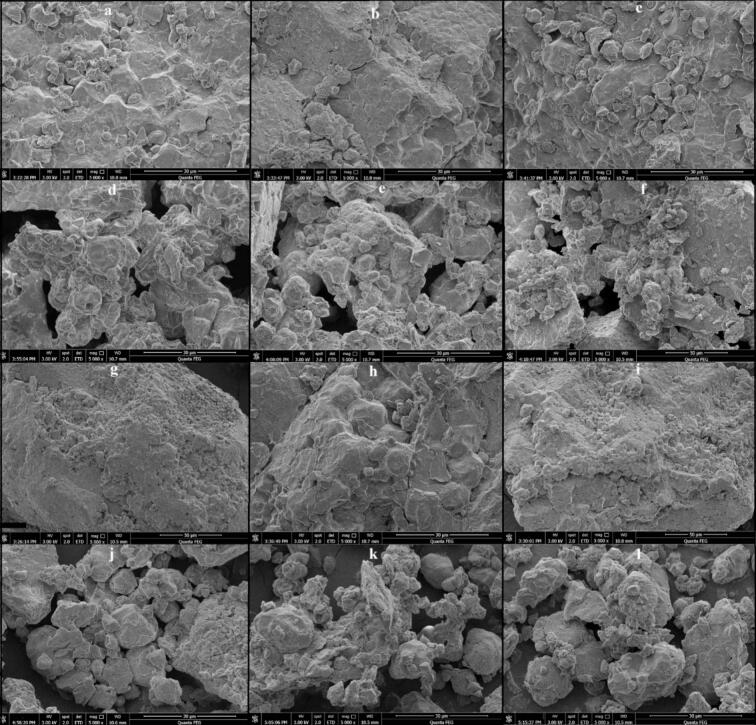
Scanning electron micrographs of (Se-GBR) and enzymatic black rice (Se-GEBR) under different drying treatments.

SEM micrographs revealed distinct modifications in the microstructure of selenium-enriched black rice under different drying methods (Fig. 4a-c). In HAD, grains exhibited compaction, cell collapse, and reduced porosity due to prolonged thermal exposure. In contrast, IVD maintained greater structural integrity with moderate porosity and visible microchannels, reflecting efficient infrared heating under reduced thermal load in vacuum [39]. FD showed the most preserved structure, with high porosity and minimal deformation, attributed to sublimation under low temperature and pressure that prevented collapse [40]. Ultrasound further altered the morphology (Fig. 4d-f), producing surface cracks, pores, and loosening of starch granules through cavitation-induced oscillation and asymmetric bubble collapse, which enhanced permeability, accelerated moisture migration, and reduced drying time [[41], [42], [43]]. Selenium enrichment (Fig. 4g-i) produced denser, more rigid structures than untreated samples, likely due to selenium incorporation into cell wall components that increased rigidity and reduced flexibility [4]. Cross-sectional images confirmed that untreated samples (Fig. 4a-c,4g-i) retained compact polygonal starch granules, which limited water absorption and gelatinization, whereas ultrasound-treated samples (Fig. 4j-l) displayed expanded intergranular spaces, micropores, and partial starch disintegration, consistent with improved rehydration [44]. These disruptions reduced protein–starch interactions, promoted hydrogen bonding and gel network formation within starch matrices [8], and enhanced viscoelastic properties, water absorption, and expansion behaviour. Collectively, the results demonstrate that ultrasound-assisted drying, particularly under HAD and IVD, improved porosity and mass transfer, while FD best preserved overall integrity. Additionally, selenium enrichment imparted extra rigidity, underscoring the strong influence of the drying strategy and ultrasound on microstructural quality.

### 5.7 Phenolic contents

The phenolic and flavonoid contents of dried black rice varied considerably depending on ultrasound pretreatment, selenium enrichment, and drying method (Table 3). TPC ranged from 166.38 to 490.98 mg GAE/100 g, with the highest values in US-EBR-HAD (490.98 mg), US-BR-IVD (456.78 mg), and EBR-HAD (406.38 mg), indicating that ultrasound combined with selenium enrichment, particularly under HAD, favored phenolic retention. In contrast, US-EBR-FD (166.38 mg) and US-EBR-IVD (193.18 mg) exhibited the lowest levels, suggesting that these treatment combinations were less effective. TFC followed a similar trend, increasing from 158.15 mg in BR-HAD to 195.06 mg in US-EBR-HAD. Meanwhile, ultrasound-assisted IVD also promoted flavonoid accumulation (188.39 mg), while FD after sonication resulted in lower values (128.56 mg). The observed ranges align with earlier reports for pigmented rice, where TPC typically ranges between 200 and 600 mg GAE/100 g [2,45,46] and TFC between 120 and 270 mg CE/100 g depending on genotype and processing [45,46]. The enhancement observed under ultrasound is attributed to cavitation-induced cellular disruption, which improves solute extractability (Chemat et al., 2011). Meanwhile, selenium enrichment may stimulate phenylpropanoid and flavonoid biosynthesis by upregulating enzymes such as phenylalanine ammonia-lyase, chalcone synthase, and flavonol synthase [47]. Conversely, the reduction in some selenium–ultrasound combinations may reflect oxidative degradation triggered by cavitation-generated radicals or stress-induced metabolic suppression [48,49]. These results demonstrate that ultrasound combined with selenium enrichment under HAD and IVD enhanced both TPC and TFC, whereas FD was less effective in preserving these compounds.

**Table 3:** Effect of ultrasound-assisted drying on antioxidant indices of selenium-enriched germinated black rice.

Samples	US Time (min)	TPC mg (GAE/100 g)	TFC (mg CE/100 g)	TAC (mg CGE/100 g)	DPPH (mM TE/g)	FRAP (mM TE/g)	TCC (mg/100 g)	PPO (%)	POD (%)	BI
BR-HAD1	−	1.56^de^	1.55f	4.90^a^	1.84^abc^	1.28^f^	0.65^cd^	90.75^bc^	104.22^b^	0.17^bcd^
BR-VD2	−	2.05^b^	1.67^e^	3.58^b^	1.53^ef^	1.39^d^	1.23^a^	92.03^bc^	92.10^c^	0.24^a^
BR-FD3	−	1.91^bc^	1.73^d^	4.84^a^	1.48^f^	1.69^c^	0.63^cd^	82.62^de^	53.75^f^	0.18^bc^
US-BR-HAD4	20	1.41^def^	1.85^c^	4.84^a^	1.97^a^	1.69^c^	0.81^bc^	106.36^a^	77.63^d^	0.18^bc^
US-BR-VD5	20	2.55^a^	2.05^b^	4.75^a^	1.53^ef^	1.72^c^	0.59^d^	107.50^a^	68.22^e^	0.20^ab^
US-BR-FD6	20	1.65^cd^	1.05^h^	4.63^a^	1.63^de^	1.34^def^	0.91^b^	90.68^bc^	53.82^f^	0.18^bc^
EBR-HAD7	−	2.03^b^	2.06^b^	3.38^b^	1.74^cd^	1.99^a^	0.72b^cd^	76.20^a^	89.68^c^	0.11^e^
EBR-VD8	−	1.30^efg^	0.75^i^	3.19^b^	1.90^ab^	1.70^c^	1.13^a^	108.28^a^	95.02^c^	0.10^e^
EBR-FD9	−	1.19^fgh^	1.17^g^	3.16^b^	1.69^d^	1.31e^f^	0.35^e^	90.89^bc^	35.50^g^	0.12d^e^
US-EBR-HAD10	20	2.51^a^	2.17^a^	3.07^b^	1.51^ef^	1.90^b^	0.26^e^	96.66^b^	70.15^e^	0.14cd^e^
US-EBR-VD11	20	1.03^gh^	1.04^h^	3.23^b^	1.83^bc^	1.21^g^	0.29^e^	84.19^cd^	120.47^a^	0.08^e^
US-EBR-FD12	20	0.94^h^	1.06^h^	3.14^b^	1.92^ab^	1.35^de^	0.87^b^	87.61^cd^	53.75^f^	0.08^e^

#### 5.7.1 TAC

The TAC of selenium-enriched black rice ranged from 3.07 to 4.90 mg CGE/100 g (Table 3). The highest concentrations were observed in BR-HAD (4.90 mg), BR-FD (4.84 mg), and US-BR-HAD (4.84 mg), demonstrating that hot-air and freeze-drying were comparatively effective in retaining anthocyanins. In contrast, selenium-enriched samples exposed to ultrasound, particularly US-EBR-HAD (3.07 mg) and US-EBR-FD (3.14 mg), showed the lowest levels, suggesting that the combined stress of selenium enrichment and ultrasonic cavitation may accelerate pigment degradation. These differences can be attributed to the sensitivity of anthocyanins to combined thermal, oxidative, and mechanical stresses. Ultrasound can rupture vacuolar membranes and expose pigments to oxygen radicals generated during cavitation, accelerating anthocyanin degradation [50]. Selenium enrichment may further alter cellular redox balance, making pigments more susceptible to oxidative breakdown. In contrast, HAD at 60 °C promotes moderate thermal softening of tissues without causing extensive oxidative damage, thereby aiding in pigment retention. IVD and FD create different oxygen- and temperature-related environments that selectively impact pigment stability; however, their effects depend on the structural integrity and enzyme activity within the grains. As anthocyanins are highly prone to oxidation, any condition that enhances PPO/POD activity or disrupts pigment-containing structures tends to reduce TAC. These results align with earlier reports indicating that anthocyanins are highly thermolabile and sensitive to oxidative stress [49], with cavitation-generated radicals further contributing to their breakdown [51,52]. Although the observed values are lower than the broader range of 5–50 mg CGE/100 g reported for pigmented rice cultivars [53], the variation is consistent with genotype- and environment-dependent differences, as well as with postharvest processing effects [2]. These findings indicate that HAD and FD are relatively effective for anthocyanin preservation, selenium–ultrasound combinations.

#### 5.7.2 Antioxidant activities (DPPH and FRAP)

The antioxidant potential of selenium-enriched black rice, evaluated by DPPH and FRAP assays, ranged from 1.48 to 1.97- and 1.21–1.99-mM TE/g, respectively (Table 3). Both assays demonstrated that ultrasound-assisted treatments, particularly US-BR-HAD (1.97 and 1.69 mM TE/g) and US-EBR-HAD (1.92 and 1.90 mM TE/g), enhanced the antioxidant capacity compared to the untreated samples, reflecting a greater release of phenolics through cavitation-induced cell wall disruption [14,54]. The improvement in sonicated samples is primarily attributed to cavitation-induced rupturing of cell walls, which facilitates the release of bound phenolics and flavonoids. Furthermore, HAD promotes slight thermal softening of cellular matrices, enhancing the extractability of antioxidant compounds without causing excessive degradation. In contrast, FD and some IVD treatments resulted in lower antioxidant activity, likely due to structural collapse and reduced accessibility of phenolics trapped within freeze-dried matrices. Selenium enrichment also modulates antioxidant behavior, as selenium participates in redox reactions and may alter phenylpropanoid biosynthesis, influencing the antioxidant potential of the grains. Interestingly, US-EBR-FD showed relatively high DPPH activity despite moderate FRAP values, indicating that different antioxidant constituents may contribute variably to radical scavenging and reducing power [54]. This divergence suggests that different antioxidant pathways depend on the specific phenolic subclasses preserved under each processing condition. Treatments that retain more free phenolics tend to show more vigorous DPPH activity, whereas those preserving redox-active compounds such as flavonols contribute more to FRAP values. These observations are consistent with earlier studies, which report that ultrasound pretreatment enhances antioxidant activity by improving the extractability of bound phenolics. Meanwhile, selenium enrichment may contribute additional metabolites that interact with redox systems [55,56]. Collectively, the results demonstrate that ultrasound-assisted HAD and IVD are the most effective strategies for enhancing the antioxidant properties of selenium-enriched black rice.

#### 5.7.3 TCC

The total carotenoid content (TCC) of selenium-enriched black rice ranged between 0.26 and 1.23 mg/100 g (d.w.). The highest concentrations were recorded in BR-IVD (1.23 mg) and EBR-IVD (1.13 mg), indicating that vacuum drying was more effective in preserving carotenoids than hot-air or freeze-drying. This enhanced stability can be attributed to the reduced oxygen environment and lower effective heat load in IVD, which limits carotenoid oxidation and isomerization. Because carotenoids contain conjugated double bonds, they are highly susceptible to thermal degradation and oxidative cleavage. IVD minimizes these reactions by decreasing oxygen availability and maintaining lower moisture levels. Conversely, ultrasound-treated selenium-enriched samples, especially US-EBR-HAD (0.26 mg) and US-EBR-IVD (0.29 mg), showed marked decreases in carotenoids, possibly due to enhanced oxidative stress or disruption of chromoplast membranes caused by ultrasound-induced mechanical forces. FD preserved carotenoids moderately well; however, ice crystal formation and tissue collapse may expose pigments to oxidative reactions during rehydration, explaining why FD did not outperform IVD [48,57]. These results highlight the high sensitivity of carotenoids to both oxidative and thermal stress. Similar variability has also been documented in black rice, where TCC values ranging from 15.39 to 32.86 µg/g were reported, depending on the genotype, with pigmented cultivars exhibiting considerably higher levels than non-pigmented rice [58].

### 5.8 Enzyme activities

The oxidative enzyme activities of selenium-enriched black rice varied widely depending on ultrasound pretreatment and drying method (Table 3). PPO activity ranged from 76.20 % to 108.28 %, with the highest values observed in EBR-IVD (108.28 %) and US-BR-IVD (107.50 %), followed by US-BR-HAD (106.36 %). This indicates that vacuum and hot-air drying, particularly when assisted by ultrasound, stimulate PPO through enhanced substrate accessibility and redox modulation. In contrast, EBR-HAD (76.20 %) and EBR-FD (82.62 %) showed markedly lower activities, suggesting partial enzyme inactivation under mild heating or freeze-drying [6,10]. POD activity showed a broader range (35.50–120.47 %), with the maximum recorded in US-EBR-HAD (120.47 %) and BR-HAD (104.22 %), highlighting the strong stimulatory effect of ultrasound-assisted hot-air drying. This increase is consistent with the role of POD in peroxide detoxification under oxidative stress (Liao et al., 2020). Intermediate activities were observed in EBR-IVD (95.02 %), EBR-HAD (89.68 %), and BR-IVD (92.10 %). In comparison, the lowest were measured in freeze-dried samples such as US-EBR-FD (35.50 %) and BR-FD (53.75 %), reflecting cold-induced denaturation and reduced cellular permeability. PPO and POD showed parallel responses, with ultrasound and moderate-heat drying enhancing activity, and freeze-drying limiting it, confirming earlier observations that these enzymes are susceptible to processing-driven metabolic adjustments [6,7].

#### 5.8.1 Browning index (BI)

The BI of selenium-enriched black rice varied between 0.08 and 0.24 (Table 3). The highest values were observed in BR-IIVD (0.24), EBR-IIVD (0.20), and BR-FD (0.18), indicating that vacuum and freeze-drying promoted greater browning, likely through oxidative and Maillard reactions under low-moisture conditions. In IVD, low-moisture environments can accelerate non-enzymatic browning reactions such as the Maillard reaction and sugar–protein interactions, contributing to elevated BI. FD preserves endogenous enzymes such as PPO and POD, which may react upon rehydration or during sample handling, resulting in increased browning intensity. In contrast, the lowest BI was recorded in US-EBR-HAD (0.08) and US-EBR-FD (0.08), suggesting that ultrasound-assisted drying effectively limited pigment loss and enzymatic browning. This indicates that ultrasound pretreatment suppressed browning by disrupting enzyme structures or reducing substrate availability, thereby limiting pigment formation [6]. Selenium enrichment may also modulate BI by enhancing antioxidant activity or altering redox status, reducing the accumulation of brown pigments [59]. Consistently low BI values in selenium-enriched, ultrasound-treated samples such as US-EBR-IVD (0.10) support the synergistic influence of both factors in preserving visual quality. The overall BI pattern closely mirrors the trends in PPO and POD activity, emphasizing the central role of oxidative enzymes and drying conditions in browning development.

## 6 9 selenium content

**Fig. 5:**
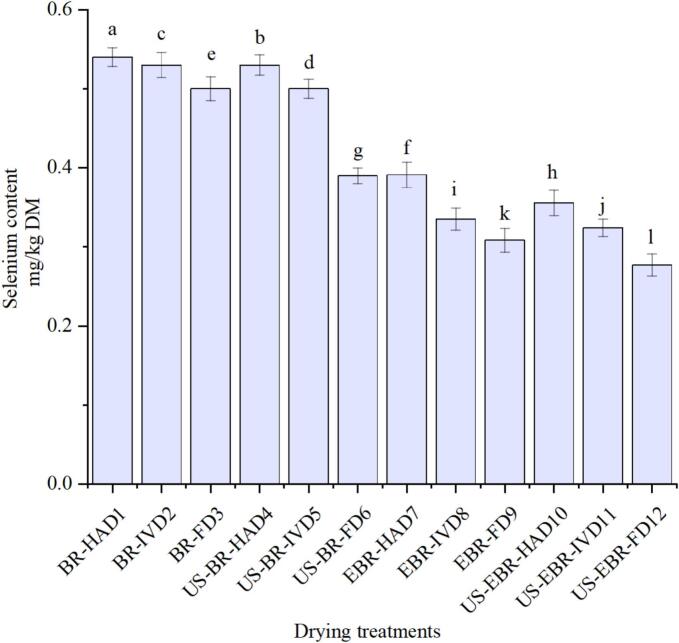
Selenium content of selenium-enriched black rice under different drying treatments.

The selenium concentrations of the twelve treatments ranged between 0.28 and 0.54 mg/kg DM, showing apparent effects of ultrasound pretreatment and drying method (Fig. 5). In the RBR group, selenium was highest in HAD1 (0.54 mg/kg) and IVD2 (0.53 mg/kg), while FD3 contained slightly lower levels (0.50 mg/kg). Ultrasound application under hot-air (US-RBR-HAD4, 0.53 mg/kg) and vacuum drying (US-RBR-IVD5, 0.50 mg/kg) yielded comparable values to those of the untreated controls. In contrast, the combination of ultrasound and freeze drying (US-RBR-FD6) markedly reduced selenium to 0.39 mg/kg. In comparison, EBR samples consistently showed lower concentrations, ranging from 0.39 mg/kg in HAD7 to 0.31 mg/kg in FD9, with further reductions following ultrasound pretreatment, as observed in US-EBR-HAD10 (0.36 mg/kg), US-EBR-IVD11 (0.32 mg/kg), and US-EBR-FD12 (0.28 mg/kg). These results demonstrate that selenium retention is strongly influenced by processing conditions, with hot-air and vacuum drying supporting higher stability. In contrast, freeze drying, particularly when combined with ultrasound, resulted in greater losses. Similar outcomes have been reported previously, where moderate thermal drying preserved selenoamino acids more effectively, whereas freeze drying promoted redistribution or volatilization due to sublimation-related structural changes [1,60]. The disruptive effect of ultrasound on cellular microstructure has also been shown to increase mineral mobility, which could explain the pronounced decline under freeze drying [61]. From a nutritional perspective, the selenium concentrations obtained in this study fall within the internationally recommended range for cereals (0.3–0.8 mg/kg dry matter, DM), ensuring that a 100 g serving would supply approximately 28–54 µg of selenium. This intake is sufficient to meet or closely approach the adult daily requirement of 55 µg, while remaining well below the tolerable upper intake level of 400 µg/day established by EFSA and FDA [62,63]. Comparable values have been achieved in earlier biofortification studies, where selenium levels in rice and wheat ranged between 0.4 and 0.7 mg/kg, thereby contributing effectively to dietary adequacy without exceeding safe thresholds [64]. The present findings indicate that selenium-enriched black rice subjected to hot-air or vacuum drying can retain concentrations aligned with nutritional guidelines, offering a safe and effective means of enhancing dietary selenium intake. In contrast, ultrasound-assisted freeze drying requires careful optimization to minimize nutrient loss.

### 6.1 HPLC analyses

Changes in phenolic acids, flavonoids, and anthocyanins during germination and subsequent drying of black rice are summarized in Table 4. A total of eleven phenolic acids, three flavonoids, and four anthocyanins were identified. The total phenolic acid content varied widely, ranging from 642.64 µg/g in US-EBR-IIVD to 2918.66 µg/g in BR-FD. Freeze-dried samples, such as BR-FD, US-BR-FD, and US-BR-HAD, consistently exhibited higher levels, reflecting minimal thermal degradation and enhanced release of bound constituents. Gallic acid was the predominant phenolic acid, reaching 1502.97 µg/g in BR-FD. In contrast, chlorogenic acid peaked in US-BR-IVD, suggesting selective preservation of hydroxycinnamic acids under vacuum-assisted sonication. Lower phenolic acid levels in selenium-enriched samples, such as US-EBR-IVD, EBR-IVD, and EBR-HAD, may indicate enzymatic degradation or downregulation of biosynthesis in response to selenium-related stress (Nguyen et al., 2023). Flavonol content followed a similar pattern, ranging from 92.00 µg/g in BR-HAD to 128.84 µg/g in US-BR-IVD, with quercetin as the dominant flavanol. This enrichment was particularly notable in ultrasound-assisted vacuum and freeze-dried treatments, consistent with sonication improving the extractability and availability of compounds such as kaempferol and rutin [65]. These findings are consistent with earlier studies showing that germination enhances phenolic accumulation through esterase activity and activation of enzymes, including phenylalanine ammonia-lyase, glucosidase, and chalcone isomerase [66,67].

**Table 4:** Effect of ultrasound-assisted drying on phenolic profile of selenium-enriched germinated black rice.

**Compounds**	RT	**Concentration range (µg. g^-1^dm)**											**ANOVA**
	(min)	BR-HAD1	BR-VD2	BR-FD3	US-BR-HAD4	US-BR-VD5	US-BR-FD6	EBR-HAD7	EBR-VD8	EBR-FD9	US-EBR-HAD10	US-EBR-VD11	
**Phenolics**													
Caffeic acid	28.23	47.79^g^	57.58^bc^	62.54^a^	48.22^g^	52.32^de^	50.50^ef^	48.54^fg^	59.42^b^	56.59^c^	52.90^d^	49.28^fg^	< 0.0001
Chlorogenic acid	13.33	41.17^g^	81.56^b^	71.85^c^	49.06^f^	89.06^a^	83.81^b^	60.83^e^	60.58^e^	63.26^d^	46.87^f^	42.27^g^	< 0.0001
Ellagic acid	13.48	182.98^g^	191.80^d^	214.23^c^	189.13^e^	187.70^f^	192.26^d^	183.75^g^	222.52^b^	235.40^a^	183.68^g^	189.38^e^	< 0.0001
Ferulic acid	13.54	54.04^a^	52.21^cd^	51.42^cd^	52.81^abc^	52.30^bcd^	52.43^bcd^	53.92^a^	51.67^cd^	51.23^d^	53.69^ab^	ND^e^	< 0.0001
Gallic acid	13.46	947.50^c^	579.82^d^	1502.97^a^	1134.70^b^	384.01^f^	295.79^g^	548.23^e^	222.29^i^	366.65^f^	264.11*^h^*	222.77^i^	< 0.0001
P-Hydroxybenzoic acid	13.32	22.40^g^	24.25^f^	2.58^j^	4.93^h^	202.59^a^	26.39^e^	36.69^b^	28.95^d^	30.66^c^	3.53^i^	28.43^d^	< 0.0001
Isoferulic acid	34.17	289.82^b^	290.87^a^	291.08^a^	290.70^a^	6.43^c^	ND^d^	291.19^a^	ND	ND	289.75^b^	ND	< 0.0001
*P*-coumaric acid	12.81	8.17^b^	6.53^d^	5.55^e^	7.25^c^	32.41^a^	ND^f^	6.46^d^	6.75^cd^	5.30^e^	8.27^b^	ND	< 0.0001
Protocatechuic acid	13.45	311.82^c^	280.52^d^	545.78^a^	383.60^b^	177.16^g^	149.17^i^	196.64^f^	123.95^j^	161.83^h^	252.41^e^	124.69^j^	< 0.0001
Sinapic acid	13.36	185.96^a^	177.05^e^	169.06^h^	181.07^c^	58.01*^h^*	175.89^ef^	184.34^b^	174.81^fg^	173.94^g^	182.28^c^	179.07^d^	< 0.0001
Vanillic acid	13.09	45.83^e^	53.17^c^	1.60^g^	32.48^f^	ND	47.21^d^	67.87^a^	44.90^e^	53.88^c^	68.12^a^	57.95^b^	< 0.0001
**Total phenolic acids**		2137.48	1795.36	2918.66	2373.95	1241.99	1073.45	1678.46	995.84	1198.74	1405.61	642.64	
**Flavanols**													
Kaempferol	12.77	48.94^e^	56.14^a^	53.06^c^	48.41^e^	53.29^c^	55.13^b^	51.89^d^	56.05^ab^	53.24^c^	52.04^d^	56.28^a^	< 0.0001
Quercetin	22.57	38.99^g^	49.19^c^	55.26^b^	41.08^f^	64.96^a^	43.85^e^	45.99^d^	43.11^e^	64.88^a^	49.23^c^	43.86^e^	< 0.0001
Rutin	27.11	4.07^f^	8.41^bc^	8.30^c^	4.36^f^	10.59^a^	8.78b^c^	5.48^e^	7.70^d^	8.85^b^	7.37^d^	5.68^e^	< 0.0001
**Total Flavonols**		92.00	113.74	116.62	93.85	128.84	107.76	103.36	106.86	126.97	108.64	105.82	
**Anthocyanins**	13.58												
Cyanidin-3-galactoside	15.38	359.70^g^	429.08^a^	429.17^a^	366.29^f^	ND	406.60^b^	361.02^g^	400.67^c^	395.85^d^	ND	383.00^e^	< 0.0001
Cyanidin-3-glucoside	17.60	242.19^j^	2746.15^b^	1488.02^h^	239.07^j^	3075.64^a^	2105.29^f^	1963.47^g^	2417.50^d^	2311.83^e^	905.13^i^	2577.69^c^	< 0.0001
Cyanidin-3- diglucoside	12.72	196.59^h^	265.70^e^	409.22^a^	197.56^h^	262.51^f^	266.11^de^	300.99^b^	267.81^cd^	267.10^cde^	203.14^g^	268.70^c^	< 0.0001
Cyanidin-3-Rhamnoglucoside	13.34	2.62^g^	18.33^b^	31.78^a^	5.25^f^	16.38^c^	17.78^b^	3.16^g^	16.12^c^	15.14^d^	5.34^f^	10.53^e^	< 0.0001
**Total Anthocyanins**		1001.38	4324.08	2947.74	1010.21	4472.71	3494.73	3285.80	3877.63	3737.40	1484.81	4049.90	

Anthocyanins showed pronounced variability across treatments. Cyanidin-3-glucoside was the predominant anthocyanin, ranging from 627.18 µg/g in BR-HAD to 3075.64 µg/g in US-BR-IVD, confirming that ultrasound-assisted vacuum drying improved retention of this thermolabile compound. Similar findings were reported for black rice anthocyanins [2]. The lowest cyanidin-3-glucoside content (627.18 µg/g) in BR-HAD highlights its sensitivity to hot-air drying, consistent with earlier evidence that anthocyanins are highly unstable under heat and oxidative stress (Hou et al., 2013). Decomposition of cyanidin-3-glucoside into protocatechuic acid and phloroglucinaldehyde [68] further explains the observed decline under thermal conditions. Cyanidin-3-diglucoside varied from 167.87 µg/g in US-EBR-FD to 1094.89 µg/g in US-BR-IVD. In comparison, cyanidin-3-rhamnoglucoside ranged between 82.18 and 231.52 µg/g, with both compounds exhibiting the highest values in ultrasound-assisted IVD, suggesting greater stability under oxygen-limited drying conditions. In contrast, cyanidin-3-galactoside was consistently the least abundant, ranging from 45.93 to 106.79 µg/g, but its relative stability across treatments indicates resilience to processing stress. Selenium-enriched and ultrasound-treated samples, particularly US-EBR-FD and US-EBR-IVD, exhibited significant anthocyanin losses, which may be attributed to pigment leaching during ultrasonication or structural weakening due to cavitation [2]. These findings highlight that while freeze-drying maximized phenolic acid retention, ultrasound-assisted IVD provided the greatest protection for anthocyanins, especially cyanidin-3-glucoside. In contrast, selenium enrichment introduced additional instability, underscoring the importance of carefully optimizing combined treatments to preserve polyphenolic quality in black rice.

### 6.2 Volatile compounds

**Fig. 6:**
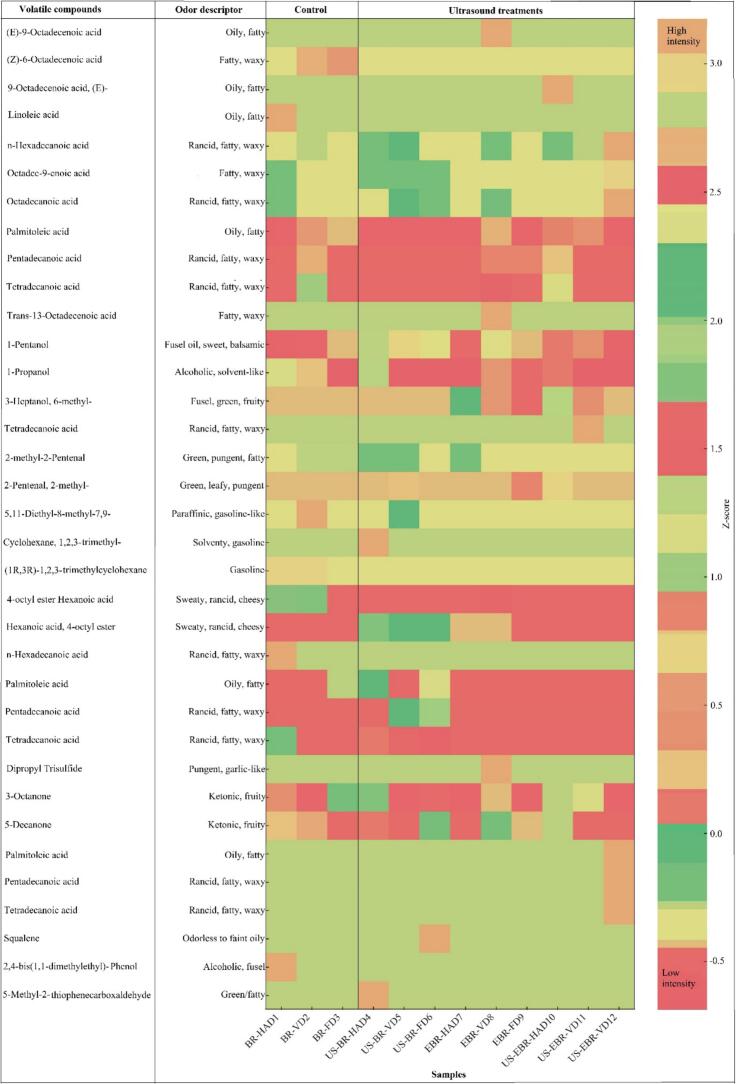
Heatmap of volatile compounds in selenium-biofortified black rice subjected to different treatments.

Volatile compounds play a central role in the sensory acceptance of black rice, and their profiles were strongly affected by ultrasound pretreatment and drying method. GC–MS identified 38 volatiles across diverse classes, including 11 acids, 5 alcohols, 2 aldehydes, 7 esters, 5 ketones, 4 alkanes, and minor phenols, olefins, and sulfur derivatives (supplementary Table S3 and S4), consistent with earlier observations that aldehydes, alcohols, ketones, and esters dominate the volatile profile of pigmented rice [1]. Ultrasound-assisted drying enhanced the formation and retention of desirable compounds while suppressing aldehydes linked with rancid or beany notes. Alcohols showed marked increases under ultrasound, particularly 3-heptanol (6-methyl), which reached 8.784 ng/g (57.93 % relative content) in US-EBR-HAD, and 1-hexanol, which increased to 3.26 ng/g in US-BR-FD, contributing grassy and fruity aroma characteristics [69]. Acids such as n-hexadecanoic acid (0.929 ng/g, 2.7 %) and octadec-9-enoic acid (0.482 ng/g) were most abundant in US-EBR-IVD, reflecting reduced oxidative loss under vacuum and cavitation-enhanced release from cell matrices, in line with Deng et al. (2022) [15]. Aldehydes showed the opposite trend, being abundant in conventionally dried samples but diminished with ultrasound. BR-HAD contained 2-methyl-2-pentenal at 4.47 % of total volatiles, a compound with pungent, rancid notes associated with linoleic acid degradation. Still, its abundance decreased to 0.26 % in US-BR-FD and 0.43 % in US-EBR-FD. Hexanal, a key contributor to beany flavor, also declined in ultrasound-assisted treatments, confirming suppression of lipid oxidation under cavitation and oxygen-limiting conditions [7,70]. This reduction in aldehydes aligns with earlier reports that ultrasound limits secondary oxidation pathways, while moderate heating may deactivate lipoxygenase and promote Maillard reactions, thereby altering carbonyl profiles [71]. Ketones were enhanced in specific treatments, with 3-octanone rising to 1.02 ng/g in US-BR-FD, imparting mushroom-like notes through oxidative and enzymatic lipid cleavage facilitated by ultrasound. Esters were detected at trace levels (0.001–0.037 ng/g, ≤0.067 %) but were slightly higher in US-EBR-HAD and US-BR-IIVD, indicating limited esterification between alcohols and fatty acids promoted by cavitation [72]. Hydrocarbons such as hexadecane and 1,2,3-trimethylcyclohexane were present at very low levels (<0.03 %) across all treatments. Their negligible contribution to aroma is consistent with their high odor thresholds, though their presence indicates thermal degradation of waxes or lipid cleavage [73]. Importantly, ultrasound did not promote hydrocarbon accumulation, underscoring its mild, non-thermal character. The heatmap provided a clear visualization of these variations, where ultrasound-assisted treatments exhibited stronger intensities of alcohols and fatty acids. At the same time, aldehydes associated with rancid notes became more prominent in the control group (Fig. 6). This graphical distribution supports the quantitative findings, highlighting that ultrasound pretreatment and the drying method jointly shaped the volatile profile of selenium-enriched black rice. Overall, ultrasound-assisted HAD and IVD enhanced alcohols and fatty acids while reducing aldehydes and preserving carotenoid-derived volatiles, thereby improving the sensory profile of selenium-enriched black rice. These outcomes support earlier findings that ultrasound stabilizes volatiles in cereal matrices by enhancing mass transfer, creating microchannels, and reducing oxidative degradation under limited oxygen exposure [1,69].

### 6.3 Multivariate analysis of antioxidant indices and phenolic compounds

**Fig. 7:**
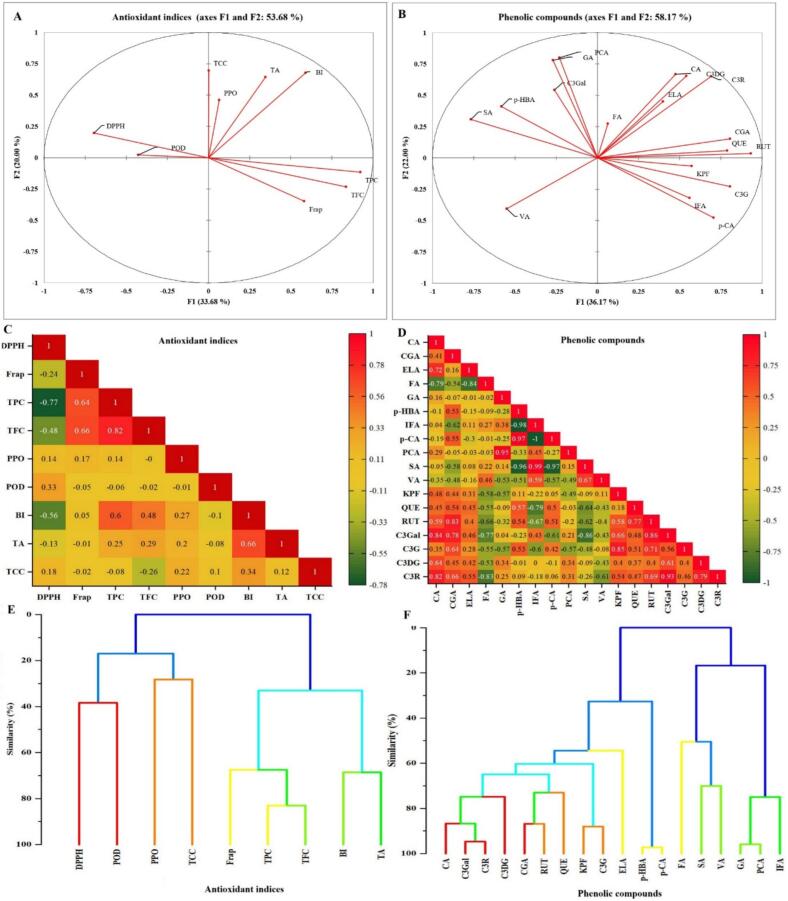
Multivariate analysis of antioxidant indices and phenolic compounds in selenium-biofortified black rice: (A, B) principal component analysis (PCA); (C, D) correlation heatmaps; (E, F) hierarchical cluster analysis (HCA).

The multivariate evaluation of antioxidant indices and phenolic compounds in selenium-enriched black rice highlighted distinct associations among biochemical traits. In Fig. 7a, the PCA of antioxidant indices explained 53.68 % of the total variance, with TPC, TFC, and FRAP firmly aligned along PC1, confirming the central role of phenolic content in determining reducing capacity. In contrast, PPO and POD were positioned in the opposite direction, indicating an antagonistic effect of oxidative enzymes on antioxidant stability through degradation of phenolic substrates. At the same time, BI and TA showed independent contributions. The PCA of phenolic compounds (Fig. 7b) accounted for 58.17 % of the variance, where flavonoids such as quercetin, rutin, catechin derivatives, and chlorogenic acid clustered closely, reflecting their shared biosynthetic pathway. In contrast, hydroxybenzoic acids (gallic, syringic, vanillic, and p-hydroxybenzoic acids) contributed more distinctly to PC2, suggesting pathway-specific regulation. These associations were further supported by correlation analysis (Fig. 7c-d), which showed strong positive relationships among TPC, TFC, and FRAP but weak or negative correlations of PPO and POD with antioxidant indices, while phenolic compounds grouped according to their structural classes, with flavonoids highly intercorrelated and benzoic acids more independent. Cluster analysis (Fig. 7e-f) confirmed these patterns, with FRAP, TPC, and TFC forming one branch, PPO and POD clustering separately, and BI and TA aligning together, indicating a distinction between phenolic-driven antioxidant mechanisms and physicochemical attributes. For phenolics, flavonoids, and chlorogenic acid, a cluster was formed, while benzoic acids and syringic acid formed separate branches. Collectively, these multivariate results demonstrate that phenolic composition, particularly flavonoids, exerts a dominant influence on antioxidant potential. In contrast, oxidative enzymes negatively regulate antioxidant stability, and browning-related indices contribute independently to sample differentiation, thereby elucidating the biochemical basis underlying the observed treatment effects in black rice.

## 7 Conclusion

This study demonstrates the effectiveness of ultrasound-assisted drying in enhancing both processing efficiency and functional quality of selenium-enriched germinated black rice. Ultrasound combined with hot-air or infrared vacuum drying increased moisture diffusivity, reduced energy demand, and shortened drying time by up to 16.7 %. The Hii model provided an excellent fit for the drying kinetics. Cavitation-induced microchannels and surface disruptions facilitated moisture transport, contributing to improved drying behaviour. Phytochemical retention was markedly enhanced, with total phenolics and flavonoids being significantly higher under ultrasound-assisted hot-air and vacuum drying. In contrast, freeze-drying was more effective in preserving individual phenolic acids. Anthocyanins, particularly cyanidin-3-glucoside, were best retained in ultrasound-assisted vacuum drying, whereas carotenoid stability was maximized under vacuum drying without ultrasound. SEM confirmed that ultrasound-treated grains exhibited structural loosening and porosity, consistent with enhanced mass transfer. GC–MS profiling further showed enrichment of alcohols and organic acids, alongside a reduction in aldehydes associated with off-flavors. Collectively, these findings validate ultrasound-assisted HAD and IVD as promising non-thermal strategies for enhancing efficiency, preserving bioactivity, and improving aroma quality in functional cereal products.

## CRediT authorship contribution statement

**Muhammad Tayyab Rashid:** Writing – original draft, Methodology, Formal analysis. **Kunlun Liu:** Supervision. **Nazish Muzaffar:** Software. **Basim M. Alohali:** Visualization, Data curation. **Mushtaque Ahmed Jatoi:** Writing – review & editing. **Hazrat Usman:** Visualization. **Rana Muhammad Aadil:** Writing – review & editing, Visualization.

## Declaration of competing interest

The authors declare that they have no known competing financial interests or personal relationships that could have appeared to influence the work reported in this paper.
