# Supplementary material for: Impact of ultrasound-assisted germination and varied drying methods on phenolic biosynthesis, antioxidant capacity, and oxidative enzyme activity in selenium-biofortified black rice
Source: Ultrason Sonochem. 2025 Nov 23;123:107692. doi: 10.1016/j.ultsonch.2025.107692 (PMC12686892; doi:10.1016/j.ultsonch.2025.107692)
Supplement: Supplementary Data 1 [file mmc1.docx]

**Supplementary Table S1**. Drying models used to study drying kinetic of black rice.

| **S. No** | **Model name** | **Model** | **References** |
| --- | --- | --- | --- |
| 1 | Newton | $Mr=exp(-kt)$ | [1] |
| 2 | Page | $Mr=exp(-kt^{n}$) | [2] |
| 3 | Henderson and Pabis model | $Mr=aexp(-kt^{n}$) | [3] |
| 4 | Modified Henderson and Pabis | $Mr=aexp\left( -kt \right)+bexp\left( -gt \right)+cexp(-ht)$ | [4] |
| 5 | Logarithmic model | $Mr=aexp\left( -kt \right)+c$ | [5] |
| 6 | Midilli model | $Mr=aexp\left( -kt \right)+bt$ | [6] |
| 7 | Two-term model | $Mr=aexp\left( {-k}_{1}t \right)+bexp\left( {-k}_{2}t \right)$ | [7] |
| 8 | Hii model | $Mr=aexp\left( {-k}_{1}t^{n} \right)+bexp\left( {-k}_{2}t^{n} \right)$ | [8] |
| 9 | Verma model | $Mr=aexp\left( -kt \right)+\left( 1-a \right)exp(-gt)$ | [9] |
| 10 | Modified Midilli model | $Mr=aexp\left( -kt \right)+b$ | [10] |
| 11 | Aghbashlo model | $Mr=exp\left( k_{1}t/1+k_{2}t \right)$ | [11] |
| 12 | Wang and Singh | $Mr=1+at+bt^{2}$ | [12] |
| 13 | Silva model | $Mr=exp(-at-b\sqrt{t})$ | [13] |

**Supplementary Table S2.** Averages of selected models fitted to the ultrasound-treated selenium-enriched germinated black rice.

| Sample Codes | Coefficients | R^2^ | RSS | χ^2^ | RMSE |
| --- | --- | --- | --- | --- | --- |
|  | 1. Two-term model |  |  |  |  |
| RBR-HAD1 RBR-VD2 US-RBR-HAD4 US-RBR-VD5 EBR-HAD7 EBR-VD8 US-EBR-HAD10 US-EBR-VD11 | a. 0.4850, k1. 0.2400, b. 0.5010, k2. 0.0030  a. 0.9810, k1. 0.0110, b. 0.0490, k2. 0.0060 a. 0.7590, k1. 0.0110, b. 0.2350, k2. 0.0010 a. 0.8900, k1. 0.0090, b. 0.1250, k2. 0.0020 a. 0.4610, k1. 0.0480, b. 0.5320, k2. 0.0020 a. 0.6920, k1. 0.2000, b. 0.3110, k2, 0.0000 a. 0.5140, k1. 0.4200, b. 0.4820, k2. 0.0020 a. 0.6330, k1. 0.0290, b. 0.3680, k2. 0.0000 | 0.9940 0.9810 0.9990 0.9930 0.9890 0.9990 0.9970 0.9990 | 0.0000 0.0100 0.0200 0.0000 0.0000 0.0000 0.0000 0.0000 | 0.0002 0.0001 0.0020 0.0003 0.0005 5.5900×10^-05^ 0.0001 2.9600×10^-05^ | 0.0152 0.0113 0.0395 0.0192 0.0183 0.0079 0.0113 0.0057 |
|  | 2, Midilli Model | R^2^ | RSS | χ^2^ | RMSE |
| RBR-HAD1 RBR-VD2 US-RBR-HAD4 US-RBR-VD5 EBR-HAD7 EBR-VD8 US-EBR-HAD10 US-EBR-VD11 | a. 0.0000, k. -9.3100, n. 0.0320, b. 0.0010  a. 0.7610, k. 0.0620, n. -4.9100×10^-008^, b. 0.0002 a. 0.8400, k. 0.0250, n. -3.7300×10^-008^, b. 0.0030 a. 0.7600, k. -4.2280, n. -1.6100, b. 0.0010 a. 0.0000, k. -8.3320, n. 0.0330, b. -6.2800×10^-005^ a. 0.0020, k. -7.8750, n. 0.0890, b. 0.0000 a. 0.0000, k. -8.9740, n. 0.0410, b. -4.4400×10^-005^ a. 0.2350, k. -7.2060, n. 0.5830, b. 0.0000 | 0.9810 1.0000 0.9490 0.9990 0.9730 0.9990 0.9920 1.0000 | 0.0000 0.0600 0.0100 0.0000 0.0000 0.0000 0.0000 0.0000 | 0.0002 0.0092 0.0014 2.6900×10^-05^ 0.0003 2.6500×10^-05^ 7.9800×10^-05^ 7.5800×10^-05^ | 0.0150 0.0849 0.0327 0.0057 0.0148 0.0054 0.0089 0.0029 |
|  | 3, Verma Model | R^2^ | RSS | χ^2^ | RMSE |
| RBR-HAD1 RBR-VD2 US-RBR-HAD4 US-RBR-VD5 EBR-HAD7 EBR-VD8 US-EBR-HAD10 US-EBR-VD11 | a. 0.4340, k. 0.0070, g. 0.0070  a. 0. 380, k. 0.0060, g. 0.0060 a. 0.3430, k. 0.0060, g. 0.0060 a. 0.3800, k. 0.0060, g. 0.0060 a. 0.3620, k. 0.0070, g. 0.0070 a. 0.4100, k. 0.0080, g. 0.0080 a. 0.4090, k. 0.0080, g. 0.0080 a. 0.3650, k. 0.0070, g. 0.0070 | 0.8750 0.9730 0.9530 0.9730 0.5450 0.8590 0.6950 0.6340 | 0.0400 0.0300 0.0500 0.0100 0.1100 0.0500 0.1200 0.1100 | 0.0038 0.0046 0.0054 0.0007 0.0175 0.0059 0.0110 0.0124 | 0.0568 0.0602 0.0639 0.0302 0.1082 0.0817 0.1053 0.1185 |
|  | 4, Henderson and Pabis model | R^2^ | RSS | χ^2^ | RMSE |
| RBR-HAD1 RBR-VD2 US-RBR-HAD4 US-RBR-VD5 EBR-HAD7 EBR-VD8 US-EBR-HAD10 US-EBR-VD11 | a. 1.0030, k. 0.0520, n. 0.5940  a. 1.0220, k. 0.0290, n. 0.7210 a. 1.0080, k. 0.0230, n. 0.7230 a. 1.0150, k. 0.0150, n. 0.8140 a. 1.0020, k. 0.1440, n. 0.3650 a. 1.0050, k. 0,068, n. 0.5420 a. 1.0030, k.0.1240, n. 0,426 a. 1.0020, k. 0.1650, n. 0.3140 | 0.9950 0.9480 0.9950 0.9860 0.9910 0.9830 0.9930 0.9750 | 0.0000 0.0200 0.0100 0.0100 0.0000 0.0100 0.0000 0.0100 | 0.0001 0.0034 0.0011 0.0005 0.0003 0.0008 0.0002 0.0008 | 0.0113 0.0519 0.0298 0.0256 0.0156 0.0302 0.0159 0.0316 |
|  | 5, Logarithmic model | R^2^ | RSS | χ^2^ | RMSE |
| RBR-HAD1 RBR-VD2 US-RBR-HAD4 US-RBR-VD5 EBR-HAD7 EBR-VD8 US-EBR-HAD10 US-EBR-VD11 | a. 0.6970, k. 0.0150, c. 0.2760  a. 0.7810, k. 0.0150, c.0.2540 a. 0.7090, k. 0.0120, c. 0.2850 a. 0.7850, k. 0.0100, c. 0.2310 a. 0.5820, k. 0.0280, c. 0.3940 a. 0.6840, k. 0.0200, c. 0.3190 a. 0.6390, k. 0.0280, c. 0.3450 a. 0.6090, k. 0.0310, c. 0.3930 | 0.9930 0.9770 0.9990 0.9990 0.9930 0.9990 0.9920 0.9990 | 0.0000 0.0100 0.0200 0.0000 0.0100 0.0000 0.0000 0.0000 | 0.0003 0.0020 0.0019 0.0003 0.0010 5.5900×10^-05^ 0.0003 4.5200×10^-05^ | 0.0179 0.0394 0.0386 0.0192 0.0261 0.0079 0.0187 0.0079 |
|  | 6, Aghbashlo Model | R^2^ | RSS | χ^2^ | RMSE |
| RBR-HAD1 RBR-VD2 US-RBR-HAD4 US-RBR-VD5 EBR-HAD7 EBR-VD8 US-EBR-HAD10 US-EBR-VD11 | k1. 0.4120, k2. 0.4200  k1. 1.0690, k2. -1.0760  k1. -2.6360, k2. 2.6300 k1. 0.2860, k2. 0.2920 k1.1.3890, k2. -1.3960 k1.1.1366, k2. -1.3730 k1. 0.5310, k2. 0.5390 k1. 0.1970, k2. 0.1890 | 0.8750 0.9140 0.9530 0.9730 0.5450 0.8590 0.6950 0.6340 | 0.0400 0.0300 0.0500 0.0100 0.1100 0.0500 0.1200 0.1100 | 0.0038 0.0046 0.0054 0.0007 0.0175 0.0059 0.0110 0.0124 | 0.0568 0.0602 0.0639 0.0302 0.1082 0.0817 0.1053 0.1185 |
|  | 7, Wang and Singh | R^2^ | RSS | χ^2^ | RMSE |
| RBR-HAD1 RBR-VD2 US-RBR-HAD4 US-RBR-VD5 EBR-HAD7 EBR-VD8 US-EBR-HAD10 US-EBR-VD11 | a. 0.0080, b. 2.0810  a. 0. 0.0080, b. 2.0700×10^-005^ a. 0.0070, b. 1.7100×10^-005^ a. 0.0060, b. 1.3400×10^-005^ a. 0.0090, b. 2.7600×10^-005^ a. 0.0090, b. 2.7600×10^-005^ a. 0.0100, b. 3.4200×10^-005^ a. 0.0090, b. 3.1500×10^-005^ | 0.9480 0.9750 0.9910 0.9890 0.8170 0.9620 0.8720 0.8980 | 0.0200 0.0100 0.0300 0.0000 0.0500 0.0100 0.0500 0.0300 | 0.0020 0.0017 0.0036 0.0003 0.0081 0.0016 0.0047 0.0034 | 0.0417 0.0372 0.0523 0.0215 0.0738 0.0430 0.0687 0.0624 |
|  | 8, Silva Model | R^2^ | RSS | χ^2^ | RMSE |
| RBR-HAD1 RBR-VD2 US-RBR-HAD4 US-RBR-VD5 EBR-HAD7 EBR-VD8 US-EBR-HAD10 US-EBR-VD11 | a. 0.0020, b. 0.0640  a. 0.004, b. 0.0390 a. 0.0030, b. 0.0330 a. 0.0040, b. 0.0190 a. 0.0020, b. 0.1030 a. 0.0000, b. 0.7800 a. 0.0020, b. 0.1060 a. 0.0030, b. 0.1130 | 0.9930 0.0940 0.9920 0.9830 0.9950 0.9820 0.9950 0.9840 | 0.0000 0.0300 0.0100 0.0100 0.0000 0.0100 0.0000 0.0100 | 0.0001 0.0041 0.0010 0.0005 0.0002 0.0007 0.0001 0.0004 | 0.0116 0.0570 0.0280 0.0254 0.0133 0.0250 0.0135 0.0236 |
|  | 9, Newton | R^2^ | RSS | χ^2^ | RMSE |
| RBR-HAD1 RBR-VD2 US-RBR-HAD4 US-RBR-VD5 EBR-HAD7 EBR-VD8 US-EBR-HAD10 US-EBR-VD11 | k. 0.0070 k. 0.0070 k. 0.0060 k. 0.0060 k. 0.0070 k. 0.0080 k. 0.0080 k. 0.0070 | 0.8750 0.9140 0.9530 0.9730 0.5450 0.8590 0.6950 0.6340 | 0.0400 0.0300 0.0500 0.0100 0.1100 0.0500 0.1200 0.1100 | 0.0038 0.0046 0.0054 0.0007 0.0175 0.0059 0.0110 0.0124 | 0.0568 0.0602 0.0639 0.0302 0.1082 0.0817 0.1053 0.1185 |
|  | 10. Page | R^2^ | RSS | χ^2^ | RMSE |
| RBR-HAD1 RBR-VD2 US-RBR-HAD4 US-RBR-VD5 EBR-HAD7 EBR-VD8 US-EBR-HAD10 US-EBR-VD11 | k. 0.0510, n. 0.5970 k. 0.0250, n. 0.7400 k. 0.0220, n. 0.7320 k. 0.0130, n. 0.8330 k. 0.1430, n. 0.3660 k. 0.0670, n. 0.5450 k. 0.1230, n. 0.4280 k. 0.1640, n. 0.3420 | 0.9950 0.9470 0.9950 0.9850 0.9910 0.9830 0.9930 0.9750 | 0.0000 0.0300 0.0100 0.0100 0.0000 0.0100 0.0000 0.0100 | 0.0001 0.0035 0.0111 0.0005 0.0003 0.0008 0.0002 0.0008 | 0.0113 0.0527 0.0296 0.0254 0.0156 0.0316 0.0159 0.0316 |

**Supplementary Table S3.** Effect of ultrasound and different drying methods on selenium-enriched germinated black rice regarding volatile compounds.

|  |  |  | Control | | | Ultrasound-treated samples (ng/g) | | | | | | | | |
| --- | --- | --- | --- | --- | --- | --- | --- | --- | --- | --- | --- | --- | --- | --- |
| **No.** | **Compounds** | RT | BR-HAD1 | BR-VD2 | BR-FD3 | US-BR-HAD4 | US-BR-VD5 | US-BR-FD6 | EBR-HAD7 | EBR-VD8 | EBR-FD9 | US-EBR-HAD10 | US-EBR-VD11 | US-EBR-VD12 |
| **A** | **Acids** |  |  |  |  |  |  |  |  |  |  |  |  |  |
| 1 | (E)-9-Octadecenoic acid | 48.77 | - | - | - | - | - | - | - | 0.030 | - | - | - | - |
| 2 | (Z)-6-Octadecenoic acid | 48.77 | - | 0.007 | 0.002 | - | - | - | - | - | - | - | - | - |
| 3 | 9-Octadecenoic acid, (E)- | 48.77 | - | - | - | - | - | - | - | - | - | 0.019 | - | - |
| 4 | Linoleic acid | 48.67 | 0.006 | - | - | - | - | - | - | - | - | - | - | - |
| **5** | n-Hexadecanoic acid | 45.52 | 0 | 0.027 | 0.003 | 0.033 | 0.080 | 0.007 | 0.000 | 0.056 | 0.002 | 0.038 | 0.029 | 0.929 |
| 6 | Octadec-9-enoic acid | 48.76 | 0.017 | - | - | 0.029 |  | 0.027 | - | - | - | - | - | 0.482 |
| **7** | Octadecanoic acid | 49.20 | 0.007 | - | - | 0 | 0.012 | 0.007 | - | 0.009 | - | - | - | 0.124 |
| 8 | Palmitoleic acid | 49.05 | - | 0.007 | 0.001 | - | - | - | - | 0.019 | 0.000 | 0.009 | 0.006 | - |
| 9 | Pentadecanoic acid | 43.49 | - | 0.017 | - | - | - | - | - | 0.007 | - | 0.004 | - | - |
| 10 | Tetradecanoic acid | 41.44 | - | 0.006 | - | - | - | - | - | 0.012 | 0.000 | 0.007 | - | - |
| 11 | trans-13-Octadecenoic acid | 48.79 | - | - | - | - | - | - | - | 0.030 | - | - | - | - |
| **B** | **Alcohols** |  |  |  |  |  |  |  |  |  |  |  |  |  |
| 12 | 1-Pentanol | 3.82 | - | 0.003 | 0.025 | 0.426 | 0.172 | 0.027 | 0.008 | 0.033 | 0.026 | 0.083 | 0.123 | - |
| 13 | 1-Propanol | 1.45 | 0.098 | 0.028 | - | 0.067 | - | - | 0.001 | 0.042 | 0.004 | 0.026 | - | - |
| 14 | 2-Heptanol, 2-methyl- | 12.11 | - | - | - | - | - | - | 0.000 | - | - | - | - | - |
| 15 | 3-Heptanol, 6-methyl- | 20.15 | - | - | - | - | - | - | 0.929 | - | - | 8.784 | 2.075 | - |
| 16 | Tetradecanoic acid | 41.44 | - | - | - | - | - | - | - | - | - | - | 0.005 | - |
| **C** | **Aldehyde** |  |  |  |  |  |  |  |  |  |  |  |  |  |
| 17 | 2-methyl-2-Pentenal | 5.82 | - | 0.762 | - | 0.051 | - | - | 0.051 | - | - | - | - | - |
| 18 | 2-Pentenal, 2-methyl- | 5.80 | - | - | - | - | 0.007 | - | - | - | 0.012 | 0.032 | - | - |
| **D** | **Alkane** |  |  |  |  |  |  |  |  |  |  |  |  |  |
| 19 | 5,11-Diethyl-8-methyl-7,9-dioxapentadecane | 39.75 | - | 0.027 | - | - | 0.002 | - | - | - | - | - | - | - |
| 20 | Hexadecane | 37.73 | - | - | - | - | - | - | 0.000 | - | 0.000 | - | - | - |
| 21 | Cyclohexane, 1,2,3-trimethyl-, (1.alpha.,2.alpha.,3.beta.)- | 27.51 | - | - | - | 0.017 | - | - | - | - | - | - | - | - |
| 22 | (1R,3R)-1,2,3-trimethylcyclohexane | 28.35 | 0.027 | - | - | - | - | - | - | - | - | - | - | - |
| **E** | **Ester** |  |  |  |  |  |  |  |  |  |  |  |  |  |
| 23 | 4-octyl ester Hexanoic acid | 34.82 | 0.028 | 0.029 | - | - | - | - | - | 0.026 | - | - | - | - |
| 24 | Hexanoic acid, 4-octyl ester | 34.83 | - | - | - | 0.032 | 0.037 | 0.007 | 0.001 | - | - | - | - | - |
| 25 | n-Hexadecanoic acid | 45.50 | 0.029 | - | - | - | - | - | - | - | - | - | - | - |
| 26 | Palmitoleic acid | 45.07 | - | - | - | 0.004 | 0.019 | 0.027 | - | - | - | - | - | - |
| 27 | Pentadecanoic acid | 43.50 | - | - | 0.000 | - | 0.008 | 0.007 | - | - | - | - | - | - |
| 28 | pentyl ester-Pentanoic acid | 24.81 | - | - | 0.000 | - | - | - | - | - | - | - | - | - |
| 29 | Tetradecanoic acid | 41.42 | 0.003 | - | - | 0.005 | 0.018 | 0.027 | - | - | - | - | - | - |
| **F** | **Ethers** |  |  |  |  |  |  |  |  |  |  |  |  |  |
| 30 | dipropyl Trisulfide | 30.21 | - | - | - | - | - | - | - | 0.001 | - | - | - | - |
| **G** | **Ketone** |  |  |  |  |  |  |  |  |  |  |  |  |  |
| 31 | 3-Octanone | 14.26 | 0.762 | 0.019 | 0.351 | 1.883 | - | 0.059 | - | 0.148 | - | 0.250 | 2.326 | - |
| 32 | 5-Decanone | 25.21 | 0.019 | 0.098 | - | 0.015 | - | 0.007 | 0.000 | 0.007 | 0.001 | 0.005 | - | - |
| 33 | Palmitoleic acid | 45.07 | - | - | - | - | - | - | - | - | - | - | - | 0.224 |
| 34 | Pentadecanoic acid | 43.07 | - | - | - | - | - | - | - | - | - | - | - | 0.115 |
| 35 | Tetradecanoic acid | 41.42 | - | - | - | - | - | - | - | - | - | - | - | 0.194 |
| **H** | **Olefins** |  |  |  |  |  |  |  |  |  |  |  |  |  |
| 36 | Squalene | 53.79 | - | - | - | - | - | 0.027 | - | - | - | - | - | - |
| **I** | **Phenols** |  |  |  |  |  |  |  |  |  |  |  |  |  |
| 37 | 2,4-bis(1,1-dimethylethyl)-Phenol | 35.67 | 0.394 | - | - | - | - | - | - | - | - | - | - | - |
| **J** | **Thiophene** |  |  |  |  |  |  |  |  |  |  |  |  |  |
| 38 | 5-Methyl-2-thiophenecarboxaldehyde thiosemicarbazone | 26.03 | - | - | - | 0.010 | - | - | - | - | - | - | - | - |

Note. "–" means not detected

**Supplementary Table S4**. Effect of ultrasound and different drying methods on the relative content (%) of selenium-enriched germinated black rice regarding volatile compounds.

|  |  |  | Control | | | Ultrasound-treated samples (%) | | | | | | | | |
| --- | --- | --- | --- | --- | --- | --- | --- | --- | --- | --- | --- | --- | --- | --- |
| **No.** | **Compounds** | RT | BR-HAD1 | BR-VD2 | BR-FD3 | US-BR-HAD4 | US-BR-VD5 | US-BR-FD6 | EBR-HAD7 | EBR-VD8 | EBR-FD9 | US-EBR-HAD10 | US-EBR-VD11 | US-EBR-VD12 |
| **A** | **Acids** |  |  |  |  |  |  |  |  |  |  |  |  |  |
| 1 | (E)-9-Octadecenoic acid | 48.77 | - | - | - | - | - | - | - | 0.016 | - | - | - | - |
| 2 | (Z)-6-Octadecenoic acid | 48.77 | - | 0.016 | 0.006 | - | - | - | - | - | - | - | - | - |
| 3 | 9-Octadecenoic acid, (E)- | 48.77 | - | - | - | - | - | - | - | - | - | 0.002 | - | - |
| 4 | Linoleic acid | 48.67 | 0.001 | - | - | - | - | - | - | - | - | - | - | - |
| **5** | n-Hexadecanoic acid | 45.52 | - | 0.023 | 0.008 | 0.012 | 0.035 | 0.013 | 0.026 | 0.031 | 0.009 | 0.004 | 0.006 | 0.027 |
| 6 | Octadec-9-enoic acid | 48.76 | 0.002 | - | - | 0.010 | - | 0.011 | - | - | - | - | - | 0.014 |
| **7** | Octadecanoic acid | 49.20 | 0.001 | - | 0.001 | - | 0.005 | 0.001 | - | 0.005 | - | - | - | 0.004 |
| 8 | Palmitoleic acid | 49.05 | - | 0.005 | 0.002 | - | - | - | - | 0.010 | 0.002 | 0.001 | 0.001 | - |
| 9 | Pentadecanoic acid | 43.49 | - | 0.002 | - | - | - | - | - | 0.004 | - | 0.000 | - | - |
| 10 | Tetradecanoic acid | 41.44 | - | 0.005 | - | - | - | - | - | 0.007 | 0.001 | 0.001 | - | - |
| 11 | trans-13-Octadecenoic acid | 48.79 | - | - | - | - | 0.029 | - | - | - | - | - | - | - |
| **B** | **Alcohols** |  |  |  |  |  |  |  |  |  |  |  |  |  |
| 12 | 1-Pentanol | 3.82 | - | 0.007 | 0.062 | 0.152 | 0.075 | 0.093 | 0.517 | 0.018 | 0.145 | 0.009 | 0.027 | - |
| 13 | 1-Propanol | 1.45 | 0.010 | 0.025 | - | 0.024 | - | - | 0.088 | 0.022 | 0.023 | 0.003 | - | - |
| 14 | 2-Heptanol, 2-methyl- | 12.11 | - | - | - | - | - | - | 0.006 | - | - | - | - | - |
| 15 | 3-Heptanol, 6-methyl- | 20.15 | - | - | - | - | - | - | 57.93 | - | - | 0.951 | 0.452 | - |
| 16 | Tetradecanoic acid | 41.44 | - | - | - | - | - | - | - | - | - | - | 0.001 | - |
| **C** | **Aldehyde** |  |  |  |  |  |  |  |  |  |  |  |  |  |
| 17 | 2-methyl-2-Pentenal | 5.82 | - | 0.017 | - | 0.018 | - | - | 0.028 | - | - | - | - | - |
| 18 | 2-Pentenal, 2-methyl- | 5.80 | - | - | - | - | 0.019 | - | - | - | 0.001 | 0.007 | - | - |
| **D** | **Alkane** |  |  |  |  |  |  |  |  |  |  |  |  |  |
| 19 | 5,11-Diethyl-8-methyl-7,9-dioxapentadecane | 39.75 | - | 0.003 | - | - | 0.001 | - | - | - | - | - | - | - |
| 20 | Hexadecane | 37.73 | - | - | - | - | - | - | 0.004 | - | 0.001 | - | - | - |
| 21 | Cyclohexane, 1,2,3-trimethyl-, (1.alpha.,2.alpha.,3.beta.)- | 27.51 | - | - | - | 0.006 | - | - | - | - | - | - | - | - |
| 22 | (1R,3R)-1,2,3-trimethylcyclohexane | 28.35 | 0.003 | - | - | - | - | - | - | - | - | - | - | - |
| **E** | **Ester** |  |  |  |  |  |  |  |  |  |  |  |  |  |
| 23 | 4-octyl ester Hexanoic acid | 34.82 | 0.003 | 0.028 | - | - | - | - | - | 0.014 | - | - | - | - |
| 24 | Hexanoic acid, 4-octyl ester | 34.83 | - | - | - | 0.011 | 0.016 | 0.014 | 0.067 | - | - | - | - | - |
| 25 | n-Hexadecanoic acid | 45.50 | 0.003 | - | - | - | - | - | - | - | - | - | - | - |
| 26 | Palmitoleic acid | 45.07 | - | - | - | 0.001 | 0.008 | 0.003 | - | - | - | - | - | - |
| 27 | Pentadecanoic acid | 43.50 | - | - | 0.001 | - | 0.003 | 0.001 | - | - | - | - | - | - |
| 28 | pentyl ester-Pentanoic acid | 24.81 | - | - | - | - | - | - | - | - | - | - | - | - |
| 29 | Tetradecanoic acid | 41.42 | 0.000 | - | 0.002 | 0.002 | 0.008 | 0.003 | - | - | - | - | - | - |
| **F** | **Ethers** |  |  |  |  |  |  |  |  |  |  |  |  |  |
| 30 | dipropyl Trisulfide | 30.21 | - | - | - | - | - | - | - | 0.001 | - | - | - | - |
| **G** | **Ketone** |  |  |  |  |  |  |  |  |  |  |  |  |  |
| 31 | 3-Octanone | 14.26 | 0.079 | 0.864 | 0.888 | 0.820 | - | 3.682 | - | 0.814 | - | 0.027 | 0.506 | - |
| 32 | 5-Decanone | 25.21 | 0.002 | 0.003 | - | 0.006 | - | 0.004 | 0.014 | 0.004 | 0.004 | 0.001 | - | - |
| 33 | Palmitoleic acid | 45.07 | - | - | - | - | - | - | - | - | - | - | - | 0.007 |
| 34 | Pentadecanoic acid | 43.07 | - | - | - | - | - | - | - | - | - | - | - | 0.003 |
| 35 | Tetradecanoic acid | 41.42 | - | - | - | - | - | - | - | - | - | - | - | 0.006 |
| **H** | **Olefins** |  |  |  |  |  |  |  |  |  |  |  |  |  |
| 36 | Squalene | 53.79 | - | - | - | - | - | 0.001 | - | - | - | - | - | - |
| **I** | **Phenols** |  |  |  |  |  |  |  |  |  |  |  |  |  |
| 37 | 2,4-bis(1,1-dimethylethyl)-Phenol | 35.67 | 0.001 | - | - | - | - | - | - | - | - | - | - | - |
| **J** | **Thiophene** |  |  |  |  |  |  |  |  |  |  |  |  |  |
| 38 | 5-Methyl-2-thiophenecarboxaldehyde thiosemicarbazone | 26.03 | - | - | 0.003 | - | - | - | - | - | - | - | - | - |

*Note. "–" means not detected.*

**References**

[1] A. El-Beltagy, G.R. Gamea, A.H.A. Essa, Solar drying characteristics of strawberry, J. Food Eng. 78 (2007) 456–464. https://doi.org/10.1016/j.jfoodeng.2005.10.015.

[2] E.O.M. Akoy, Experimental characterization and modeling of thin-layer drying of mango slices, Int. Food Res. J. 21 (2014) 1911–1917.

[3] N. Hashim, O. Daniel, E. Rahaman, A Preliminary Study: Kinetic Model of Drying Process of Pumpkins (Cucurbita Moschata) in a Convective Hot Air Dryer, Agric. Agric. Sci. Procedia 2 (2014) 345–352. https://doi.org/10.1016/j.aaspro.2014.11.048.

[4] M.S. Zenoozian, H. Feng, S.M.A. Razavi, F. Shahidi, H.R. Pourreza, Image analysis and dynamic modeling of thin-layer drying of osmotically dehydrated pumpkin, J. Food Process. Preserv. 32 (2008) 88–102. https://doi.org/10.1111/j.1745-4549.2007.00167.x.

[5] K. Kulwinder, S. A.K., Drying kinetics and quality characteristics of beetroot slices under hot air followed by microwave finish drying, African J. Agric. Res. 9 (2014) 1036–1044. https://doi.org/10.5897/AJAR2013.

[6] M. Campus, A.- Bellevue, Mathematical modeling of thin layer drying kinetics of apples slices, Int. Food Res. J. 19 (2006) 1949–1958. https://doi.org/10.1051/IUFoST.

[7] K. Sacilik, Effect of drying methods on thin-layer drying characteristics of hull-less seed pumpkin (Cucurbita pepo L.), J. Food Eng. 79 (2007) 23–30. https://doi.org/10.1016/j.jfoodeng.2006.01.023.

[8] C.L. Hii, C.L. Law, M. Cloke, Modeling using a new thin layer drying model and product quality of cocoa, J. Food Eng. 90 (2009) 191–198. https://doi.org/10.1016/j.jfoodeng.2008.06.022.

[9] K. An, D. Zhao, Z. Wang, J. Wu, Y. Xu, G. Xiao, Comparison of different drying methods on Chinese ginger (Zingiber officinale Roscoe): Changes in volatiles, chemical profile, antioxidant properties, and microstructure, Food Chem. 77 (2016) 4716–4724. https://doi.org/10.1016/j.phytochem.2015.07.012.

[10] P.L. Gan, P.E. Poh, Investigation on the Effect of Shapes on the Drying Kinetics and Sensory Evaluation Study of Dried Jackfruit, Int. J. Sci. Eng. 7 (2014) 193–198. https://doi.org/10.12777/ijse.7.2.193-198.

[11] İ. Doymaz, S. Karasu, M. Baslar, Effects of infrared heating on drying kinetics, antioxidant activity, phenolic content, and color of jujube fruit, J. Food Meas. Charact. 10 (2016) 283–291. https://doi.org/10.1007/s11694-016-9305-4.

[12] A.O. Omolola, A.I.O. Jideani, P.F. Kapila, Modeling microwave drying kinetics and moisture diffusivity of mabonde banana variety, Int. J. Agric. Biol. Eng. 7 (2014) 107–113. https://doi.org/10.3965/j.ijabe.20140706.013.

[13] W.P. da Silva, C.M.D.P.S. e Silva, F.J.A. Gama, J.P. Gomes, Mathematical models to describe thin-layer drying and to determine drying rate of whole bananas, J. Saudi Soc. Agric. Sci. 13 (2014) 67–74. https://doi.org/10.1016/j.jssas.2013.01.003.
